# Bounds on entanglement dimensions and quantum graph parameters via noncommutative polynomial optimization

**DOI:** 10.1007/s10107-018-1287-z

**Published:** 2018-05-21

**Authors:** Sander Gribling, David de Laat, Monique Laurent

**Affiliations:** 1 CWI, Amsterdam, Netherlands; 2 Tilburg University, Tilburg, Netherlands

**Keywords:** Entanglement dimension, Polynomial optimization, Quantum graph parameters, Quantum correlation, 05C15, 81P40, 81P45, 90C22, 90C26, 90C30

## Abstract

In this paper we study optimization problems related to bipartite quantum correlations using techniques from tracial noncommutative polynomial optimization. First we consider the problem of finding the minimal entanglement dimension of such correlations. We construct a hierarchy of semidefinite programming lower bounds and show convergence to a new parameter: the minimal average entanglement dimension, which measures the amount of entanglement needed to reproduce a quantum correlation when access to shared randomness is free. Then we study optimization problems over synchronous quantum correlations arising from quantum graph parameters. We introduce semidefinite programming hierarchies and unify existing bounds on quantum chromatic and quantum stability numbers by placing them in the framework of tracial polynomial optimization.

## Introduction

### Bipartite quantum correlations

One of the distinguishing features of quantum mechanics is quantum entanglement, which allows for nonclassical correlations between spatially separated parties. In this paper we consider the problems of quantifying the advantage entanglement can bring (first investigated through Bell inequalities in the seminal work [3]) and quantifying the minimal amount of entanglement necessary for generating a given correlation (initiated in [5] and continued, e.g., in [43, 53, 60]).

Quantum entanglement has been widely studied in the bipartite correlation setting (for a survey, see, e.g., [44]). Here we have two parties, Alice and Bob, where Alice receives a question *s* taken from a finite set *S* and Bob receives a question *t* taken from a finite set *T*. The parties do not know each other’s questions, and after receiving the questions they do not communicate. Then, according to some predetermined protocol, Alice returns an answer *a* from a finite set *A* and Bob returns an answer *b* from a finite set *B*. The probability that the parties answer (*a*, *b*) to questions (*s*, *t*) is given by a *bipartite correlation* *P*(*a*, *b*|*s*, *t*), which satisfies $P(a,b|s,t) \ge 0$ for all $(a,b,s,t)\in \varGamma$ and $\sum _{a,b} P(a,b|s,t) = 1$ for all $(s,t)\in S\times T$. We set $\varGamma =A\times B \times S \times T$ throughout. Which bipartite correlations $P=(P(a,b|s,t))\in \smash {\mathbb {R}^\varGamma }$ are possible depends on the additional resources available to the two parties Alice and Bob.

When the parties do not have access to additional resources the correlation *P* is *deterministic*, which means it is of the form $P(a,b|s,t) = P_A(a|s) \, P_B(b|t)$ for all $(a,b,s,t)\in \varGamma$, where $\smash {P_A=(P_A(a|s))}$ and $\smash {P_B=(P_B(b|t))}$ take their values in $\{0,1\}$ and satisfy

1 $$\begin{aligned} \sum _a P_A(a|s) = \sum _b P_B(b|t) = 1 \quad \text {for all} \quad (s,t)\in S\times T. \end{aligned}$$

When the parties use local randomness the above functions $P_A$ and $P_B$ are convex combinations of 0 / 1-valued ones, that is, $P_A$ and $P_B$ take their values in [0, 1] and satisfy (1).

When the parties have access to shared randomness the resulting correlation *P* is a convex combination of deterministic correlations and *P* is said to be a *classical correlation*. The classical correlations form a polytope, denoted $C_{loc}(\varGamma )$, whose valid inequalities are known as *Bell inequalities* [3].

We are interested in the quantum setting, where the parties have access to a shared quantum state upon which they can perform measurements. The quantum setting can be modeled in different ways, leading to the so-called tensor model and commuting model; see the discussion, e.g., in [12, 36, 58].

In the *tensor model*, Alice and Bob each have access to “one half” of a finite dimensional *quantum state*, which is modeled by a unit vector $\psi \in \mathbb {C}^d \otimes \mathbb {C}^d$ (for some $d\in \mathbb {N}$). Alice and Bob determine their answers by performing a measurement on their part of the state. Such a measurement is modeled by a *positive operator valued measure* (POVM), which consists of a set of $d \times d$ Hermitian positive semidefinite matrices labeled by the possible answers and summing to the identity matrix. If Alice uses the POVM $\{E_s^a\}_{a \in A}$ when she gets question $s \in S$ and Bob uses the POVM $\{F_t^b\}_{b \in B}$ when he gets question $t \in T$, then the probability of obtaining the answers (*a*, *b*) is given by

2 $$\begin{aligned} P(a,b|s,t) = \mathrm {Tr}( (E_s^a \otimes F_t^b) \psi \psi ^*) = \psi ^* (E_s^a \otimes F_t^b) \psi . \end{aligned}$$

If the state $\psi$ can be written as $\psi =\psi _A \otimes \psi _B$, then $P(a,b|s,t)= (\psi _A^*E^a_s\psi _A) (\psi _B^*F^b_t\psi _B)$ for all (*a*, *b*, *s*, *t*), and thus *P* is a classical correlation. Otherwise, $\psi$ is said to be *entangled* and can be used to produce a nonclassical correlation *P*.

A correlation of the above form (2) is called a *quantum correlation*; it is said to be *realizable in the tensor model in local dimension* *d* (or in *dimension* $d^2$) when $\psi \in \mathbb {C}^d\otimes \mathbb {C}^d$ and $E^a_s,F^b_t\in \mathbb {C}^{d\times d}$. Let $\smash {C_{q}^d(\varGamma )}$ be the set of such correlations and define

$$\begin{aligned} C_{q}(\varGamma )=\bigcup _{d\in \mathbb {N}} C_{q}^{d}(\varGamma ). \end{aligned}$$

Denote the smallest dimension needed to realize $P \in C_{q}(\varGamma )$ in the tensor model by

3 $$\begin{aligned} D_q(P) = \min \big \{ d^2 : d \in \mathbb {N}, \, P \in C_{q}^d(\varGamma )\big \}. \end{aligned}$$

The set $C_{q}^1(\varGamma )$ contains the deterministic correlations.^1^ Hence, by Carathéodory’s theorem, $C_{loc}(\varGamma )\subseteq C_{q}^c(\varGamma )$ holds for $c = |\varGamma |+1-|S||T|$; that is, quantum entanglement can be used as an alternative to shared randomness. If *A*, *B*, *S*, and *T* all contain at least two elements, then Bell [3] shows the inclusion $C_{loc}(\varGamma ) \subseteq C_{q}(\varGamma )$ is strict; that is, quantum entanglement can be used to obtain nonclassical correlations.

The second commonly used model to define quantum correlations is the *commuting model* (or *relativistic field theory model*). Here a correlation $P\in \mathbb {R}^\varGamma$ is called a *commuting quantum correlation* if it is of the form

4 $$\begin{aligned} P(a,b|s,t) = \mathrm {Tr}(X_s^a Y_t^b \psi \psi ^*) = \psi ^* (X_s^a Y_t^b) \psi , \end{aligned}$$

where $\{X_s^a\}_a$ and $\{Y_t^b\}_b$ are POVMs consisting of bounded operators on a separable Hilbert space *H*, satisfying $[X_s^a, Y_t^b] = X_s^a Y_t^b - Y_t^bX_s^a = 0$ for all $(a,b,s,t)\in \varGamma$, and where $\psi$ is a unit vector in *H*. Such a correlation is said to be *realizable in dimension *$d = \mathrm {dim}(H)$ *in the commuting model*. Denote the set of such correlations by $C_{qc}^d(\varGamma )$ and set $C_{qc}(\varGamma ) =C_{qc}^\infty (\varGamma )$. The smallest dimension needed to realize a quantum correlation $P \in C_{qc}(\varGamma )$ is given by

5 $$\begin{aligned} D_{qc}(P) = \min \big \{ d \in \mathbb {N}\cup \{\infty \} : P \in C_{qc}^d(\varGamma )\big \}. \end{aligned}$$

If $P\in C^d_q(\varGamma )$ has a decomposition (2) with $d \times d$ matrices $E^a_s,F^b_t$, then *P* has a decomposition (4) with $d^2\times d^2$ matrices $X^a_s=E^a_s\otimes I$ and $Y^b_t=I\otimes F^b_t$. This shows the inclusion $C_{q}^d(\varGamma ) \subseteq \smash {C_{qc}^{d^2}}(\varGamma )$, and thus

6 $$\begin{aligned} D_{qc}(P) \le D_q(P) \text { for all } P \in C_{q}(\varGamma ). \end{aligned}$$

The minimum Hilbert space dimension in which a given quantum correlation *P* can be realized quantifies the minimal amount of entanglement needed to represent *P*. Computing $D_q(P)$ is NP-hard [55], so a natural question is to find good lower bounds for the parameters $D_q(P)$ and $D_{qc}(P)$. A main contribution of this paper is proposing a hierarchy of semidefinite programming lower bounds for these parameters.

As said above we have $C_{q}^d(\varGamma ) \subseteq \smash {C_{qc}^{d^2}}(\varGamma )$. Conversely, each finite dimensional commuting quantum correlation can be realized in the tensor model, although not necessarily in the same dimension [58] (see, e.g., [12] for a proof). This shows

7 $$\begin{aligned} C_{q}(\varGamma ) = \smash {\bigcup _{d\in \mathbb {N}}} C_{qc}^{d}(\varGamma )\subseteq C_{qc}(\varGamma ). \end{aligned}$$

Using a direct sum construction one can show the sets $C_q(\varGamma )$ and $C_{qc}(\varGamma )$ are convex. Whether the two sets $C_{q}(\varGamma )$ and $C_{qc}(\varGamma )$ coincide is known as Tsirelson’s problem.

In a recent breakthrough Slofstra [54] showed that the set $C_{q}(\varGamma )$ is not closed for $|A| \ge 8$, $|B| \ge 2$, $|S| \ge 184$, $|T| \ge 235$. More recently it was shown in [14] that the same holds for $|A| \ge 2$, $|B| \ge 2$, $|S| \ge 5$, $|T| \ge 5$. Using a compactness argument one sees that the set $\smash {C_q^d(\varGamma )}$ is closed for all *d*. So, when $\smash {C_q(\varGamma )}$ is not closed, the inclusions $C^d_q(\varGamma )\subset C_q(\varGamma )$ are all strict and there is a sequence $\{P_i\} \subseteq C_{q}(\varGamma )$ with $D_q(P_i) \rightarrow \infty$. Moreover, since $C_{qc}(\varGamma )$ is closed [15, Prop. 3.4], the inclusion $C_{q}(\varGamma ) \subseteq C_{qc}(\varGamma )$ is strict, thus settling Tsirelson’s problem. Whether the closure of $C_{q}(\varGamma )$ equals $C_{qc}(\varGamma )$ for all $\varGamma$ has been shown to be equivalent to having a positive answer to Connes’ embedding conjecture in operator theory [21, 42]. This conjecture has been shown to have equivalent reformulations in many different fields; we refer to [23] for an algebraic reformulation in terms of trace positivity of noncommutative polynomials.

Further variations on the above definitions are possible. For instance, we can consider a mixed state $\rho$ (a Hermitian positive semidefinite matrix $\rho$ with ${{\mathrm{Tr}}}(\rho )=1$) instead of a pure state $\psi$, where we replace the rank 1 matrix $\psi \psi ^*$ by $\rho$ in the above definitions. By convexity this does not change the sets $C_{q}(\varGamma )$ and $C_{qc}(\varGamma )$. It is shown in [53] that this also does not change the parameter $D_q(P)$, but it is unclear whether or not $D_{qc}(P)$ might decrease. Another variation would be to use projection valued measures (PVMs) instead of POVMs, where the operators are projectors instead of positive semidefinite matrices. This again does not change the sets $C_{q}(\varGamma )$ and $C_{qc}(\varGamma )$ [40], but the dimension parameters can be larger when restricting to PVMs.

When the two parties have the same question sets ($S=T$) and the same answer sets ($A=B$), a bipartite correlation $P\in \mathbb {R}^\varGamma$ is called *synchronous* if it satisfies

$$\begin{aligned} P(a,b|s,s)=0 \quad \text {for all} \quad s\in S \quad \text {and distinct} \quad a, b \in A. \end{aligned}$$

The sets of synchronous (commuting) quantum correlations are denoted $C_{q,s}(\varGamma )$ and $C_{qc,s}(\varGamma )$, respectively. We have $C_{q,s}(\varGamma )\subseteq C_{qc,s}(\varGamma )$ and the set $C_{qc,s}(\varGamma )$ is closed. The synchronous correlation sets are already rich enough in the sense that it is still the case that Connes’ embedding conjecture holds if and only if $\mathrm {cl}(C_{q,s}(\varGamma )) = C_{qc,s}(\varGamma )$ for all $\varGamma$ [13, Thm. 3.7]. The quantum graph parameters discussed in Sect. 1.3 will be defined through optimization problems over synchronous quantum correlations.

For a synchronous quantum correlation *P* it turns out that its local dimension $D_q(P)$ is given by the factorization rank of an associated completely positive semidefinite matrix $M_P$. Recall that a matrix $M \in \mathbb {R}^{n \times n}$ is called *completely positive semidefinite* if there exist $d\in \mathbb {N}$ and $d\times d$ Hermitian positive semidefinite matrices $X_1,\ldots ,X_n$ with $M = (\mathrm {Tr}(X_iX_j))$. The minimal such *d* is its *completely positive semidefinite rank*, denoted ${\text {cpsd-rank}}(M)$. Completely positive semidefinite matrices are used in [28] to model quantum graph parameters and the ${\text {cpsd-rank}}$ is investigated in [16, 17, 49, 50]. In Sect. 2 we show the following link between synchronous quantum correlations and ${\text {cpsd-rank}}$.

#### Proposition 1

The smallest local dimension in which a synchronous quantum correlation *P* can be realized is given by the completely positive semidefinite rank of the matrix $M_P$ indexed by $S \times A$ with entries $(M_P)_{(s,a),(t,b)} = P(a,b|s,t)$ for $(a,b,s,t) \in \varGamma$. That is, $D_q(P)={\text {cpsd-rank}}(M_P)$.

In [16] we used techniques from tracial polynomial optimization to define a semidefinite programming hierarchy $\{\smash {\xi _r^\mathrm {cpsd}}(M)\}_{r \in \mathbb {N}}$ of lower bounds on ${\text {cpsd-rank}}(M)$. By the above result this hierarchy gives lower bounds on the smallest local dimension in which a synchronous correlation can be realized in the tensor model. However, as shown in [16], this hierarchy typically does not converge to ${\text {cpsd-rank}}(M)$ but instead (under a certain flatness condition) to a parameter $\smash {{\xi _{*}^{\mathrm {cpsd}}}(M)}$, which can be seen as a block-diagonal version of the completely positive semidefinite rank. This *flatness condition* is a rank stabilization condition on the optimal solution of the semidefinite program defining $\smash {{\xi _{r}^{\mathrm {cpsd}}}(M)}$; for a formal definition see (21) in Sect. 3.3.

Here we use similar techniques, now exploiting the special structure of quantum correlations, to construct a hierarchy $\{{\xi _{r}^{\mathrm {q}}}(P)\}$ of lower bounds on the minimal dimension $D_q(P)$ of any—not necessarily synchronous—quantum correlation *P*. The hierarchy converges (under flatness) to a parameter ${\xi _{*}^{\mathrm {q}}}(P)$, and using the additional structure we can show that ${\xi _{*}^{\mathrm {q}}}(P)$ is equal to an interesting parameter $A_q(P) \le D_q(P)$. This parameter describes the minimal average entanglement dimension of a correlation when the parties have free access to shared randomness; see Sect. 1.2.

In the rest of the introduction we give a road map through the contents of the paper and state the main results. We will introduce the necessary background along the way.

### A hierarchy for the average entanglement dimension

We give here an overview of the results in Sect. 3 about bounding the entanglement dimension of general (non synchronous) correlations. We are interested in the minimal entanglement dimension needed to realize a given correlation $P\in C_{q}(\varGamma )$. If *P* is deterministic or only uses local randomness, then $D_{q}(P)=D_{qc}(P)=1$. But other classical correlations (which use shared randomness) have $D_q(P) \ge D_{qc}(P) > 1$, which means the shared quantum state is used as a shared randomness resource. In [5] the concept of dimension witness is introduced, where a *d-dimensional witness* is defined as a halfspace containing $\mathrm {conv}(C_{q}^d(\varGamma ))$, but not the full set $C_{q}(\varGamma )$. As a measure of entanglement this suggests the parameter

8 $$\begin{aligned} \mathrm {inf} \Big \{ \max \nolimits _{i \in [I]} D_q(P_i) : I \in \mathbb {N},\, \lambda \in \mathbb {R}_+^I, \, \sum _{i=1}^I \lambda _i = 1,\, P = \sum _{i=1}^I \lambda _i P_i, \, P_i \in C_{q}(\varGamma ) \Big \}. \end{aligned}$$

Observe that, for a bipartite correlation *P*, this parameter is equal to 1 if and only if *P* is classical. Hence, it more closely measures the minimal entanglement dimension when the parties have free access to shared randomness. From an operational point of view, (8) can be interpreted as follows. Before the game starts the parties select a finite number of pure states $\psi _i$ ($i \in I$) (instead of a single one), in possibly different dimensions $d_i$, and POVMs $\{E_s^a(i)\}_a$, $\{F_t^b(i)\}_b$ for each $i \in I$ and $(s,t) \in S \times T$. As before, we assume that the parties cannot communicate after receiving their questions (*s*, *t*), but now they do have access to shared randomness, which they use to decide on which state $\psi _i$ to use. The parties proceed to measure state $\psi _i$ using POVMs $\{E_s^a(i)\}_a$, $\{F_t^b(i)\}_b$, so that the probability of answers (*a*, *b*) is given by the quantum correlation $P_i$. Equation (8) then asks for the largest dimension needed in order to generate *P* when access to shared randomness is free.

It is not clear how to compute (8). Here we propose a variation of (8), and we provide a hierarchy of semidefinite programs that converges to it under flatness. Instead of considering the largest dimension needed to generate *P*, we consider the *average* dimension. That is, we minimize $\sum _{i \in I} \lambda _i D_q(P_i)$ over all convex combinations $P = \sum _{i \in I} \lambda _i P_i$. Hence, the *minimal average entanglement dimension* is defined by9in the tensor model. In the commuting model, the parameter $A_{qc}(P)$ is defined by the same expression with $D_q(P_i)$ being replaced by $D_{qc}(P_i)$. Observe that we need not replace $C_{q}(\varGamma )$ by $C_{qc}(\varGamma )$ since $D_{qc}(P) = \infty$ for any $P \in C_{qc}(\varGamma ) {\setminus } C_{q}(\varGamma )$. Moreover, in view of (6), we have the inequality

10 $$\begin{aligned} A_{qc}(P)\le A_q(P) \quad \text { for all }\ P\in C_q(\varGamma ). \end{aligned}$$

It follows by convexity that for the above definitions it does not matter whether we use pure or mixed states. We show that for the average minimal entanglement dimension it also does not matter whether we use the tensor or commuting model.

#### Proposition 2

For any $P\in C_{q}(\varGamma )$ we have $A_q(P) = A_{qc}(P)$.

We have $A_q(P) \le D_q(P)$ and $A_{qc}(P) \le D_{qc}(P)$ for $P \in C_{q}(\varGamma )$, with equality if *P* is an extreme point of $C_{q}(\varGamma )$. Hence, we have $D_q(P) = D_{qc}(P)$ if *P* is an extreme point of $C_{q}(\varGamma )$. We show that the parameter $A_q(P)$ can be used to distinguish between classical and nonclassical correlations.

#### Proposition 3

For $P\in C_q(\varGamma )$ we have $A_q(P) = 1$ if and only if $P \in C_{loc}(\varGamma )$.

As mentioned before, there exist sets $\varGamma$ for which $C_{q}(\varGamma )$ is not closed [14, 54], which implies the existence of a sequence $\{P_i\} \subseteq C_{q}(\varGamma )$ such that $D_q(P) \rightarrow \infty$. We show this also implies the existence of such a sequence with $A_q(P_i) \rightarrow \infty$.

#### Proposition 4

If $C_{q}(\varGamma )$ is not closed, then there exists a sequence $\{P_i\} \subseteq C_{q}(\varGamma )$ with $A_q(P_i) \rightarrow \infty$.

Using tracial polynomial optimization we construct a hierarchy $\{{\xi _{r}^{\mathrm {q}}}(P)\}$ of lower bounds on $A_{qc}(P)$. For each $r \in \mathbb {N}$ this is a semidefinite program, and for $r = \infty$ it is an infinite dimensional semidefinite program. We further define a (hyperfinite) variation ${\xi _{*}^{\mathrm {q}}}(P)$ of ${\xi _{\infty }^{\mathrm {q}}}(P)$ by adding a finite rank constraint on the matrix variable, so that

$$\begin{aligned} {\xi _{1}^{\mathrm {q}}}(P) \le {\xi _{2}^{\mathrm {q}}}(P) \le \cdots \le {\xi _{\infty }^{\mathrm {q}}}(P) \le {\xi _{*}^{\mathrm {q}}}(P) \le A_{qc}(P). \end{aligned}$$

We do not know whether ${\xi _{\infty }^{\mathrm {q}}}(P) = {\xi _{*}^{\mathrm {q}}}(P)$ always holds. First we show that we imposed enough constraints in the bounds ${\xi _{r}^{\mathrm {q}}}(P)$ so that ${\xi _{*}^{\mathrm {q}}}(P)=A_{qc}(P)$.

#### Proposition 5

For any $P\in C_{q}(\varGamma )$ we have ${\xi _{*}^{\mathrm {q}}}(P) = A_{qc}(P)$.

Then we show that the infinite dimensional semidefinite program ${\xi _{\infty }^{\mathrm {q}}}(P)$ is the limit of the finite dimensional semidefinite programs.

#### Proposition 6

For any $P\in C_{q}(\varGamma )$ we have ${\xi _{r}^{\mathrm {q}}}(P)\rightarrow {\xi _{\infty }^{\mathrm {q}}}(P)$ as $r\rightarrow \infty$.

Finally we give a flatness criterion under which finite convergence ${\xi _{r}^{\mathrm {q}}}(P) = {\xi _{*}^{\mathrm {q}}}(P)$ holds. The definition of flatness follows later in the paper [see (21)]; here we only note that it is a rank stabilization property which is easy to check given a solution to ${\xi _{r}^{\mathrm {q}}}(P)$.

#### Proposition 7

If ${\xi _{r}^{\mathrm {q}}}(P)$ admits a $(\lceil r/3 \rceil +1)$-flat optimal solution, then we have ${\xi _{r}^{\mathrm {q}}}(P) = {\xi _{*}^{\mathrm {q}}}(P)$.

### Quantum graph parameters

Nonlocal games have been introduced in quantum information theory as abstract models to quantify the power of entanglement, in particular, in how much the sets $C_{q}(\varGamma )$ and $C_{qc}(\varGamma )$ differ from $C_{loc}(\varGamma )$. A *nonlocal game* is defined by a probability distribution $\pi :S \times T \rightarrow [0,1]$ and a predicate $f :A \times B \times S \times T \rightarrow \{0,1\}$. Alice and Bob receive a question pair $(s,t)\in S\times T$ with probability $\pi (s,t)$. They know the game parameters $\pi$ and *f*, but they do not know each other’s questions, and they cannot communicate after they receive their questions. Their answers (*a*, *b*) are determined according to some correlation $P\in \mathbb {R}^\varGamma$, called their *strategy*, on which they may agree before the start of the game, and which can be classical or quantum depending on whether *P* belongs to $C_{loc}(\varGamma )$, $C_{q}(\varGamma )$, or $C_{qc}(\varGamma )$. Then their corresponding winning probability is given by

11 $$\begin{aligned} \sum _{(s,t)\in S\times T} \pi (s,t)\sum _{(a,b)\in A\times B} P(a,b|s,t) f(a,b,s,t). \end{aligned}$$

A strategy *P* is called *perfect* if the above winning probability is equal to one, that is, if for all $(a,b,s,t)\in \varGamma$ we have

12 $$\begin{aligned} \big (\pi (s,t)>0 \quad \text { and } \quad f(a,b,s,t)=0 \big ) \quad \Longrightarrow \quad P(a,b|s,t)=0. \end{aligned}$$

Computing the maximum winning probability of a nonlocal game is an instance of linear optimization [of the function (11)] over $C_{loc}(\varGamma )$ in the classical setting, and over $C_{q}(\varGamma )$ or $C_{qc}(\varGamma )$ in the quantum setting. Since the inclusion $C_{loc}(\varGamma ) \subseteq C_{q}(\varGamma )$ can be strict, the maximum winning probability can be higher when the parties have access to entanglement; see the CHSH game [10]. In fact there are nonlocal games that can be won with probability 1 by using entanglement, but with probability strictly less than 1 in the classical setting; see the Mermin-Peres magic square game [34, 47].

The quantum graph parameters are analogues of the classical parameters defined through the coloring and stability number games as described below. These nonlocal games use the set [*k*] (whose elements are denoted as *a*, *b*) and the set *V* of vertices of a graph *G* (whose elements are denoted as *i*, *j*) as question and answer sets.

In the *quantum coloring game*, introduced in [1, 9], we have a graph $G = (V,E)$ and an integer *k*. Here we have question sets $S=T=V$ and answer sets $A=B=[k]$, and the distribution $\pi$ is strictly positive on $V \times V$. The predicate *f* is such that the players’ answers have to be consistent with having a *k*-coloring of *G*; that is, $f(a,b,i,j)=0$ precisely when ($i=j$ and $a\ne b$) or ($\{i,j\}\in E$ and $a=b$). This expresses the fact that if Alice and Bob receive the same vertex, they should return the same color and if they receive adjacent vertices, they should return distinct colors. A perfect classical strategy exists if and only if a perfect deterministic strategy exists, and a perfect deterministic strategy corresponds to a *k*-coloring of *G*. Hence the smallest number *k* of colors for which there exists a perfect classical strategy is equal to the classical chromatic number $\chi (G)$. It is therefore natural to define the quantum chromatic number as the smallest *k* for which there exists a perfect quantum strategy. Observe that such a strategy is necessarily synchronous since, in view of (12), $f(a,b,i,i)=0$ when $a\ne b$ implies $P(a,b|i,i)=0$ when $a\ne b$.

#### Definition 1

The (commuting) quantum chromatic number $\chi _q(G)$ (resp., $\chi _{qc}(G)$) is the smallest $k\in \mathbb {N}$ for which there exists a synchronous correlation $P=(P(a,b|i,j))$ in $C_{q,s}([k]^2\times V^2)$ (resp., $C_{qc,s}([k]^2\times V^2)$) such that

$$\begin{aligned} P(a,a|i,j)=0&\quad \text {for all} \quad a\in [k], \{i,j\}\in E. \end{aligned}$$

In the *quantum stability number game*, introduced in [32, 51], we again have a graph $G = (V,E)$ and $k \in \mathbb {N}$, but now we use the question set $[k]\times [k]$ and the answer set $V\times V$. The distribution $\pi$ is again strictly positive on the question set and now the predicate *f* of the game is such that the players’ answers have to be consistent with having a stable set of size *k*, that is, $f(i,j,a,b)=0$ precisely when ($a=b$ and $i\ne j$) or [$a\ne b$ and ($i=j$ or $\{i,j\}\in E$)]. This expresses the fact that when Alice and Bob receive the same index $a=b\in [k]$, they should answer with the same vertex $i=j$ of *G*, and if they receive distinct indices $a\ne b$ from [*k*], they should answer with distinct nonadjacent vertices *i* and *j* of *G*. There is a perfect classical strategy precisely when there exists a stable set of size *k*, so that the largest integer *k* for which there exists a perfect classical strategy is equal to the stability number $\alpha (G)$. Again, such a strategy is necessarily synchronous, so we get the following definition.

#### Definition 2

The (commuting) stability number $\alpha _q(G)$ (resp., $\alpha _{qc}(G)$) is the largest integer $k\in \mathbb {N}$ for which there exists a synchronous correlation $P=(P(i,j|a,b))$ in $C_{q,s}(V^2\times [k]^2)$ (resp., $C_{qc,s}(V^2\times [k]^2)$) such that

$$\begin{aligned} P(i,j|a,b)=0&\quad \text {whenever} \quad (i=j \text { or } \{i,j\}\in E) \text { and } a\ne b\in [k]. \end{aligned}$$

The classical parameters $\chi (G)$ and $\alpha (G)$ are NP-hard. The same holds for the quantum coloring number $\chi _q(G)$ [20], and also for the quantum stability number $\alpha _q(G)$ in view of the following reduction to coloring shown in [32]:

13 $$\begin{aligned} \chi _q(G)=\min \left\{ k\in \mathbb {N}: \alpha _q(G\Box K_k)=|V|\right\} . \end{aligned}$$

Here $G\Box K_k$ is the Cartesian product of the graph $G=(V,E)$ and the complete graph $K_k$. By construction we have

$$\begin{aligned} \chi _{qc}(G)\le \chi _q(G)\le \chi (G) \quad \text { and } \quad \alpha (G)\le \alpha _q(G)\le \alpha _{qc}(G). \end{aligned}$$

The separations between $\chi _q(G)$ and $\chi (G)$, and between $\alpha _q(G)$ and $\alpha (G)$, can be exponentially large in the number of vertices. This is the case for the graphs with vertex set $\{\pm 1\}^n$ for *n* a multiple of 4, where two vertices are adjacent if they are orthogonal [1, 32, 33]. It is well known that the chromatic number of a graph increases by 1 if we add a new vertex that is adjacent to all other vertices. Surprisingly, this is not true in general for the quantum chromatic number [31]. While it was recently shown that the sets $C_{q,s}(\varGamma )$ and $C_{qc,s}(\varGamma )$ can be different [14], it is not known whether there is a separation between the parameters $\chi _q(G)$ and $\chi _{qc}(G)$, and between $\alpha _q(G)$ and $\alpha _{qc}(G)$.

We now give an overview of the results of Sect. 4 and refer to that section for formal definitions. In Sect. 4.1 we first reformulate the quantum graph parameters in terms of $C^*$-algebras, which allows us to use techniques from tracial polynomial optimization to formulate bounds on the quantum graph parameters. We define a hierarchy $\{\gamma _r^\mathrm {col}(G)\}$ of lower bounds on the commuting quantum chromatic number and a hierarchy $\{\gamma _r^\mathrm {stab}(G)\}$ of upper bounds on the commuting quantum stability number. We show the following convergence results for these hierarchies.

#### Proposition 8

There is an $r_0 \in \mathbb {N}$ such that $\gamma _r^\mathrm {col}(G) = \chi _{qc}(G)$ and $\gamma _r^\mathrm {stab}(G) = \alpha _{qc}(G)$ for all $r \ge r_0$. Moreover, if $\gamma _r^\mathrm {col}(G)$ admits a flat optimal solution, then $\gamma _r^\mathrm {col}(G) = \chi _q(G)$, and if $\gamma _r^\mathrm {stab}(G)$ admits a flat optimal solution, then $\gamma _r^\mathrm {stab}(G) = \alpha _q(G)$.

Then in Sect. 4.2 we define tracial analogues $\{{\xi _{r}^{\mathrm {stab}}}(G)\}$ and $\{{\xi _{r}^{\mathrm {col}}}(G)\}$ of Lasserre type bounds on $\alpha (G)$ and $\chi (G)$ that provide hierarchies of bounds for their quantum analogues. These bounds are more economical than the bounds $\gamma ^\mathrm{col}_r(G)$ and $\gamma ^\mathrm{stab}_r(G)$ (since they use less variables) and they also permit to recover some known bounds for the quantum parameters. We show that ${\xi _{*}^{\mathrm {stab}}}(G)$, which is the parameter ${\xi _{\infty }^{\mathrm {stab}}}(G)$ with an additional rank constraint on the matrix variable, coincides with the projective packing number $\alpha _p(G)$ from [51] and that ${\xi _{\infty }^{\mathrm {stab}}}(G)$ upper bounds $\alpha _{qc}(G)$.

#### Proposition 9

We have ${\xi _{*}^{\mathrm {stab}}}(G) = \alpha _p(G)\ge \alpha _q(G)$ and ${\xi _{\infty }^{\mathrm {stab}}}(G)\ge \alpha _{qc}(G)$.

Next, we consider the chromatic number. The tracial hierarchy $\{{\xi _{r}^{\mathrm {col}}}(G)\}$ unifies two known bounds: the projective rank $\xi _f(G)$, a lower bound on the quantum chromatic number from [32], and the tracial rank $\xi _{tr}(G)$, a lower bound on the commuting quantum chromatic number from [46]. In [13, Cor. 3.10] it is shown that the projective rank and the tracial rank coincide if Connes’ embedding conjecture is true.

#### Proposition 11

We have ${\xi _{*}^{\mathrm {col}}}(G) = \xi _f(G)\le \chi _q(G)$ and ${\xi _{\infty }^{\mathrm {col}}}(G)=\xi _{tr}(G)\le \chi _{qc}(G)$.

We compare the hierarchies ${\xi _{r}^{\mathrm {col}}}(G)$ and $\gamma _r^\mathrm {col}(G)$, and the hierarchies ${\xi _{r}^{\mathrm {stab}}}(G)$ and $\gamma ^\mathrm{stab}_r(G)$. For the coloring parameters, we show the analogue of reduction (13).

#### Proposition 12

For $r\in \mathbb {N}\cup \{\infty \}$ we have $\gamma ^\mathrm{col}_r(G)= \min \{k: {\xi _{r}^{\mathrm {stab}}}(G\Box K_k)=|V|\}.$

We show an analogous statement for the stability parameters, when using the homomorphic graph product of $K_k$ with the complement of *G*, denoted here as $K_k\star G$, and the following reduction shown in [32]:

$$\begin{aligned} \alpha _q(G)=\max \{k\in \mathbb {N}: \alpha _q(K_k\star G)=k\}. \end{aligned}$$

#### Proposition 13

For $r \in \mathbb {N}\cup \{\infty \}$ we have $\gamma ^\mathrm{stab}_r(G)= \max \{k: {\xi _{r}^{\mathrm {stab}}}(K_k\star G)=k\}.$

Finally, we show that the hierarchies $\{\gamma ^\mathrm{col}_r(G)\}$ and $\{\gamma ^\mathrm{stab}_r(G)\}$ refine the hierarchies $\{{\xi _{r}^{\mathrm {col}}}(G)\}$ and $\{{\xi _{r}^{\mathrm {stab}}}(G)\}$.

#### Proposition 14

For $$r\in \mathbb {N}\cup \{\infty , *\}$$, $${\xi _{r}^{\mathrm {col}}}(G) \le \gamma ^\mathrm{col}_r(G)$$ and $$ {\xi _{r}^{\mathrm {stab}}}(G)\ge \gamma ^\mathrm{stab}_r(G)$$.

### Techniques from noncommutative polynomial optimization

In a (commutative) polynomial optimization problem we minimize a multivariate polynomial $f(x_1,\ldots ,x_n)$ over a feasible region defined by polynomial inequalities. Such a problem has the form

$$\begin{aligned} \mathrm {inf} \big \{ f(x_1,\ldots ,x_n) : \;&\, x \in \mathbb {R}^n,\, g(x_1,\ldots ,x_n) \ge 0 \text { for } g \in \mathscr {G}\big \} \end{aligned}$$

for some finite set $\mathscr {G}$ of multivariate polynomials. Lasserre [24] and Parrilo [45] introduced the moment/sum-of-squares method to solve such problems (see, e.g., [25, 27] for details). The moment method is based on the observation that the above polynomial optimization problem is equivalent to minimizing $\int f(x)d\mu (x)$ over all probability measures $\mu$ supported on the set $D(\mathscr {G}) = \{x \in \mathbb {R}^n : g(x) \ge 0 \text { for } g \in \mathscr {G}\}$. In turn, this is equivalent to minimizing *L*(*f*) over all linear functionals *L* on the space of polynomials satisfying $L(p) \ge 0$ for all polynomials *p* that are nonnegative on $D(\mathscr {G})$. To get a tractable relaxation we then consider the linear functionals *L* on the space of polynomials up to degree 2*r* and require that *L* is nonnegative on all squares $s^2$ and weighted squares $s^2g$ (for $g\in \mathscr {G}$) of degree at most 2*r*. This condition can be expressed with a polynomially sized semidefinite program for any fixed *r*. These relaxations are good in the sense that, under a mild assumption,^2^ they converge to the optimal value of the polynomial optimization problem as *r* goes to infinity.

In [37, 48] this approach has been extended to the general eigenvalue optimization problem, which is a problem of the form

$$\begin{aligned} \mathrm {inf} \big \{ \psi ^* f(X_1,\ldots ,X_n) \psi : \;&d \in \mathbb {N},\, \psi \in \mathbb {C}^d \text { unit vector},\, X_1,\ldots ,X_n \in \mathbb {C}^{d \times d},\\&g(X_1,\ldots ,X_n) \succeq 0 \text { for } g \in \mathscr {G}\big \}. \end{aligned}$$

Here, the matrix variables $X_i$ are allowed to have any dimension $d\in \mathbb {N}$ and $\{f\}\cup \mathscr {G}$ is a set of symmetric polynomials in noncommutative variables. In a tracial optimization problem, instead of minimizing the smallest eigenvalue of $f(X_1,\ldots ,X_n)$, we minimize its normalized trace $\mathrm{Tr}(f(X_1,\ldots ,X_n))/d$ (so that the identity matrix has trace one) [6–8, 22]. Such a problem has the form

$$\begin{aligned} \mathrm {inf} \big \{ \mathrm {Tr}(f(X_1,\ldots ,X_n))/d : \;&d \in \mathbb {N},\, \, X_1,\ldots ,X_n \in \mathbb {C}^{d \times d},\\&g(X_1,\ldots ,X_n) \succeq 0 \text { for } g \in \mathscr {G}\big \}, \end{aligned}$$

where the matrix variables $X_i$ may again have any dimension *d* and $\{f\}\cup \mathscr {G}$ is a set of symmetric polynomials in noncommutative variables. The moment approach for these two problems again relies on minimizing *L*(*f*), where *L* is a linear functional on the space of *noncommutative* polynomials that either models $\psi ^*f(X_1,\ldots ,X_n)\psi$ or models the *normalized trace evaluation* $\mathrm{Tr}(f(X_1,\ldots ,X_n))/d$.

Let us focus on the tracial setting which is the setting used in this paper. As in the commutative case, one obtains tractable (semidefinite programming) relaxations by requiring *L* to “behave like a trace evaluation on noncommutative polynomials of degree at most 2*r*”. Specifically, we ask *L* to be nonnegative on all Hermitian squares $s^*s$ and weighted Hermitian squares $s^*gs$ (for $g\in \mathscr {G}$) of degree at most 2*r*, and we require the new *tracial* condition $L(pq)=L(qp)$, which indeed holds for trace evaluations; see Sect. 3.3 for details. Under an analogous mild assumption, the asymptotic limit of these relaxations is well understood: we obtain a solution $(X_1,\ldots ,X_n)$ living in a $C^*$-algebra $\mathscr {A}$ equipped with a tracial state $\tau$. The question thus becomes: when can such a solution be converted into a solution to the original tracial optimization problem, i.e., to a solution living in a usual matrix algebra?

For our purposes, a $C^*$*-algebra* $\mathscr {A}$ can be defined as a norm closed $*$-subalgebra of the space ${\mathscr {B}}(H)$ of bounded operators on a complex Hilbert space *H*. Here, the involution $^*$ on $\mathscr {B}(H)$ is the usual adjoint operation, and a $*$-subalgebra is an algebra that is closed under taking adjoints. When *H* has finite dimension *d* this means $\mathscr {A}$ is a *matrix *$*$*-algebra*, i.e., $\mathscr {A}$ is a subalgebra of $\mathbb {C}^{d\times d}$ that is closed under taking complex conjugates. Examples of matrix $*$-algebras include the full matrix algebra $\mathbb {C}^{d\times d}$ or the $*$-algebra generated by given matrices $X_1,\ldots ,X_n\in \mathbb {C}^{d\times d}$, denoted $\mathbb {C}\langle X_1,\ldots ,X_n\rangle$. An algebra is called *finite dimensional* if it is finite dimensional as a vector space. Essential for understanding the asymptotic limit of the above relaxations for tracial polynomial optimization are the following results due to Artin and Wedderburn (see  [2, 59]): Any finite dimensional $C^*$-algebra is ($*$-isomorphic to) a matrix $*$-algebra containing the identity, and in turn any such matrix $*$-algebra is isomorphic to a direct sum of full matrix algebras. We record the latter result for future reference:

#### Theorem 1

([2, 59]) Let $\mathscr {A}$ be a complex matrix $*$-subalgebra of $\mathbb {C}^{d\times d}$ containing the identity. Then there exists a unitary matrix *U* and integers $K, m_k,n_k$ for $k\in [K]$ such that

$$\begin{aligned} U \mathscr {A} U^* = \bigoplus _{k=1}^K (\mathbb {C}^{n_k \times n_k} \otimes I_{m_k}) \quad \text {and} \quad d = \sum _{k=1}^K m_k n_k. \end{aligned}$$

Going back to the question above about the asymptotic limit of the relaxations to the tracial optimization problem: when the obtained solution $(X_1,\ldots ,X_n)$ lives in a *finite dimensional* $C^*$-algebra it can be converted into an optimal matrix solution to the original tracial optimization problem. As we will later see (Theorem 3) this happens when the limit linear functional *L* satisfies some finite rank condition since then *L* is a convex combination of trace evaluations at matrix tuples $(X_1,\ldots ,X_n)$ satisfying $g(X_1,\ldots ,X_n)\succeq 0$ for all $g\in \mathscr {G}$. In addition note that this may happen at a relaxation of finite order *r* when the optimal solution *L* satisfies the so-called flatness condition (see Theorems 3 and 4).

An important feature in noncommutative polynomial optimization is the dimension independence: the optimization is over all possible matrix sizes $d \in \mathbb N$. In fact, this was the original motivation in the works [36] and [12], where noncommutative polynomial optimization was first used for approximating the set $C_{qc}(\varGamma )$ of commuting quantum correlations and the maximum winning probability of nonlocal games over $C_{qc}(\varGamma )$ (and, more generally, for computing Bell inequality violations). In some applications one may want to restrict to optimizing over matrices with restricted size *d*. In [35, 38] techniques are developed that allow to incorporate this dimension restriction by suitably selecting the linear functionals *L* in a specified space; this is used to give bounds on the maximum violation of a Bell inequality in a fixed dimension. A related natural problem is to decide what is the minimum dimension *d* needed to realize a given algebraically defined object, such as a (commuting) quantum correlation *P*. Here we propose an approach based on tracial polynomial optimization: starting from the observation that the trace of the $d\times d$ identity matrix gives its size *d*, we consider the problem of minimizing *L*(1) where *L* is a linear functional now modeling the *non-normalized* matrix trace. This approach has been used in several recent works [16, 39, 57] for lower bounding factorization ranks of matrices and tensors.

## Entanglement dimension of synchronous quantum correlations

By combining the proofs from [52] (see also [32]) and [46] one can show the following link between the minimum local dimension of a synchronous correlation and the completely positive semidefinite rank of an associated completely positive semidefinite matrix.

### Proposition 1

The smallest local dimension in which a synchronous quantum correlation *P* can be realized is given by the completely positive semidefinite rank of the matrix $M_P$ indexed by $S \times A$ with entries $(M_P)_{(s,a),(t,b)} = P(a,b|s,t)$ for $(a,b,s,t) \in \varGamma$. That is, $D_q(P)={\text {cpsd-rank}}(M_P)$.

### Proof

Suppose first that $(\psi , E_s^a, F_t^b)$ is a realization of *P* in local dimension *d* as in (2). We will show $M_P$ is completely positive semidefinite with ${{\mathrm{cpsd-rank}}}_\mathbb {C}(M_P) \le d$.

Taking the Schmidt decomposition of $\psi$, there exist nonnegative scalars $\{\lambda _i\}$ and orthonormal bases $\{u_i\}$ and $\{v_i\}$ of $\mathbb {C}^d$ such that $\psi = \smash {\sum _{i=1}^d \sqrt{\lambda _i}} \, u_i \otimes v_i$.^3^ If we replace $\psi$ by $\smash {\sum _{i=1}^d \sqrt{\lambda _i}}\, v_i \otimes v_i$ and $E_s^a$ by $U E_s^a U^*$, where *U* is the unitary matrix for which $u_i = Uv_i$ for all *i*, then $(\smash {\sum _{i=1}^d \sqrt{\lambda _i}} \, u_i \otimes v_i, E_s^a, F_t^b)$ still realizes *P* and is of the same dimension *d*.

Given such a realization $(\sum _{i=1}^d \sqrt{\lambda _i}\, v_i \otimes v_i, E^a_s, F^b_t)$ of *P*, we define the matrices

$$\begin{aligned} K = \sum _{i=1}^d \sqrt{\lambda _i} \, v_iv_i^*, \quad X_s^a = K^{1/2} E_s^a K^{1/2}, \quad Y_t^b = K^{1/2} F_t^b K^{1/2}. \end{aligned}$$

By using the identities $\mathrm {vec}(K) = \psi$ and

14 $$\begin{aligned} \mathrm {vec}(K)^* (E_s^a \otimes F_t^b) \mathrm {vec}(K) = {{\mathrm{Tr}}}(K E_s^a K F_t^b) = {{\mathrm{Tr}}}(K^{1/2} E_s^a K^{1/2} K^{1/2} F_t^b K^{1/2}), \end{aligned}$$

and substituting $X^a_s=K^{1/2}E^a_sK^{1/2}$ and $Y^b_t=K^{1/2}F^b_tK^{1/2}$, we see that

15 $$\begin{aligned} P(a,b|s,t) = \langle X_s^a, Y_t^b \rangle \quad \text {for all} \quad a,b,s,t, \end{aligned}$$

and

16 $$\begin{aligned} \langle K, K \rangle = 1, \quad \sum _a X_s^a = \sum _b Y_t^b = K \quad \text {for all} \quad s, t. \end{aligned}$$

For any $s\in S$, as *P* is synchronous we have $1=\sum _{a,b} P(a,b|s,s) =\sum _{a} P(a,a|s,s)$. Then the Cauchy–Schwarz inequality gives

$$\begin{aligned} 1&= \sum _a P(a,a|s,s) = \sum _a \langle X_s^a, Y_s^a \rangle \le \sum _a \langle X_s^a, X_s^a\rangle ^{1/2} \langle Y_s^a, Y_s^a\rangle ^{1/2} \\&\le \Big ( \sum _a \langle X_s^a, X_s^a \rangle \Big )^{1/2} \Big ( \sum _a \langle Y_s^a, Y_s^a \rangle \Big )^{1/2}\\&\le \Big \langle \sum _a X_s^a, \sum _a X_s^a \Big \rangle ^{1/2} \Big \langle \sum _a Y_s^a, \sum _a Y_s^a \Big \rangle ^{1/2} = \langle K, K\rangle = 1. \end{aligned}$$

Thus all inequalities above are equalities. The first inequality being an equality shows that there exist $\alpha _{s,a} \ge 0$ such that $X_s^a = \alpha _{s,a} Y_s^a$ for all *a*, *s*. The second inequality being an equality shows that there exist $\beta _s$ such that $\Vert X_s^a\Vert = \beta _s \Vert Y_s^a\Vert$ for all *a*, *s*. Hence,

$$\begin{aligned} \beta _s \Vert Y_s^a\Vert = \Vert X_s^a\Vert = \Vert \alpha _{s,a} Y_s^a\Vert = \alpha _{s,a} \Vert Y_s^a\Vert = \alpha _{s,a} \Vert Y_s^a\Vert \quad \text {for all} \quad a,s, \end{aligned}$$

which shows $X_s^a = \beta _s Y_s^a$ for all *a*, *s*. Since $\sum _a X_s^a = K = \sum _a Y_s^a$, we have $\beta _s = 1$ for all *s*. Thus $X_s^a = Y_s^a$ for all *a*, *s*. Therefore,

$$\begin{aligned} (M_P)_{(s,a), (t,b)} = \Big \langle X_s^a, X_t^b \Big \rangle \quad \text {for all} \quad a,b,s,t, \end{aligned}$$

which shows $M_P$ is completely positive semidefinite with ${{\mathrm{cpsd-rank}}}_\mathbb {C}(M_P) \le d$.

For the other direction we suppose $\{X_s^a\}$ are smallest possible Hermitian positive semidefinite matrices such that $(M_P)_{(s,a),(t,b)} = \langle X_s^a, X_t^b \rangle$ for all *a*, *s*, *t*, *b*. Then,

$$\begin{aligned} 1 = \sum _{a,b} P(a,b|s,t) = \sum _{a,b} \Big \langle X_s^a, X_t^b \Big \rangle = \Big \langle \sum _a X_s^a, \sum _b X_t^b\Big \rangle \quad \text {for all} \quad s,t, \end{aligned}$$

which shows the existence of a matrix *K* such that $K = \sum _a X_s^a$ for all *s*. We have $\langle K, K \rangle = 1$ and thus $\mathrm {vec}(K)$ is a unit vector. Moreover, since the factorization of $M_P$ is chosen of smallest possible size, the matrix *K* is invertible. Set $E_s^a = K^{-1/2} X_s^a K^{-1/2}$ for all *s*, *a*, so that $\sum _a E_s^a = I$ for all *s*. Then, using again (14) we obtain

$$\begin{aligned} P(a,b|s,t) = (M_P)_{(s,a),(t,b)} = \langle X_s^a, X_t^b \rangle = \mathrm {vec}(K)^* (E_s^a \otimes E_t^b) \mathrm {vec}(K), \end{aligned}$$

which shows *P* has a realization of local dimension ${{\mathrm{cpsd-rank}}}_\mathbb {C}(M_P)$. $\square$

## A hierarchy for the minimal entanglement dimension

### The minimal average entanglement dimension

Here we investigate some properties of the average entanglement dimension $A_q(\cdot )$, which was introduced in Sect. 1.2 in (9). We start by showing that it does not matter whether we use the tensor model or the commuting model.

#### Proposition 2

For any $P\in C_{q}(\varGamma )$ we have $A_q(P) = A_{qc}(P)$.

#### Proof

The inequality $A_{qc}(P) \le A_q(P)$ was observed in (10). For the reverse inequality assume we have a decomposition $P = \smash {\sum _{i=1}^I} \lambda _i P_i$, which is feasible for $A_{qc}(P)$. This means we have POVMs $\{X_s^a(i)\}_a$ and $\{Y_t^b(i)\}_b$ in $\mathbb {C}^{d_i \times d_i}$ with $[X_s^a(i),Y_t^b(i)] = 0$ and unit vectors $\psi _i\in \mathbb {C}^{d_i}$ such that $P_i(a,b|s,t) = \psi _i^* X_s^a(i) Y_t^b(i) \psi _i$ for all $(a,b,s,t) \in \varGamma$ and $i\in [I]$. We will construct another decomposition of *P* which will provide a feasible solution to $A_q(P)$ with value at most $\sum _i\lambda _id_i$.

Fix some index $i\in [I]$. Applying Theorem 1 to the matrix $*$-algebra $\mathbb {C}\langle \{X^a_s(i)\}_{a,s}\rangle$ generated by the matrices $X^a_s(i)$ for $(a,s)\in A\times S$ shows that there exist a unitary matrix $U_i$ and integers^4^ $K_i,m_k,n_k$ such that

$$\begin{aligned} U_i \mathbb {C}\langle \{X_s^a(i)\}_{a,s} \rangle U_i^* = \bigoplus _{k=1}^{K_i} (\mathbb {C}^{n_k \times n_k} \otimes I_{m_k}) \quad \text {and} \quad d_i=\sum _{k=1}^{K_i} m_kn_k. \end{aligned}$$

By assumption each matrix $Y^b_t(i)$ commutes with all the matrices in $\mathbb {C}\langle \{X^a_s(i)\}_{a,s}\rangle$, and thus $U_i Y_t^b(i)U_i^*$ lies in the algebra $\bigoplus _k (I_{n_k} \otimes \mathbb {C}^{m_k \times m_k})$. Hence, we may assume

$$\begin{aligned} X_s^a(i) = \bigoplus _{k=1}^{K_i} E_s^a(i,k) \otimes I_{m_k}, \quad Y_t^b(i) = \bigoplus _{k=1}^{K_i} I_{n_k} \otimes F_t^b(i,k), \quad \psi _i = \bigoplus _{k=1}^{K_i} \psi _{i,k}, \end{aligned}$$

with $E^a_s(i,k)\in \mathbb {C}^{n_k\times n_k}$, $F^b_t(i,k)\in \mathbb {C}^{m_k\times m_k}$, and $\psi _{i,k} \in \mathbb {C}^{n_k}\otimes \mathbb {C}^{m_k}$. Then we have

$$\begin{aligned} P_i(a,b|s,t)&= \mathrm {Tr}(X_s^a(i) Y_t^b(i) \psi _i\psi _i^*)\\&=\sum _{k=1}^{K_i} \Vert \psi _{i,k}\Vert ^2\, \underbrace{\mathrm {Tr}\left( E_s^a(i,k) \otimes F_t^b(i,k) \frac{\psi _{i,k} \psi _{i,k}^*}{\Vert \psi _{i,k}\Vert ^2}\right) }_{Q_{i,k}(a,b|s,t)}, \end{aligned}$$

where $Q_{i,k}\in C_{q}(\varGamma )$. As $\sum _k \Vert \psi _{i,k}\Vert ^2 = \Vert \psi _i\Vert ^2 = 1$, we have that $P_i=\sum _k \Vert \psi _{i,k}\Vert ^2 Q_{i,k}$ is a convex combination of the $Q_{i,k}$’s.

We now show that $Q_{i,k}\in C_{q}^{\min \{m_k,n_k\}}(\varGamma )$. Consider the Schmidt decomposition $\psi _{i,k}/\Vert \psi _{i,k}\Vert = \sum _{l=1}^{\min \{m_k, n_k\}} \lambda _{i,k,l} \, v_{i,k,l} \otimes w_{i,k,l},$ where $\lambda _{i,k,l}\ge 0$ and $\{v_{i,k,l}\}_{l=1}^{n_k} \subseteq \mathbb {C}^{n_k}$ and $\{w_{i,k,l}\}_{l=1}^{m_k} \subseteq \mathbb {C}^{m_k}$ are orthonormal bases. Define unitary matrices $V_k\in \mathbb {C}^{n_k\times n_k}$ and $W_k\in \mathbb {C}^{m_k\times m_k}$ such that $V_k v_{i,k,l}$ is the *l*th unit vector in $\mathbb {R}^{n_k}$ for $1\le l\le n_k$ and $W_k w_{i,k,l}$ is the *l*th unit vector in $\mathbb {R}^{m_k}$ for $1\le l\le m_k$. Let $E_s^a(i,k)'$ (resp., $F_t^b(i,k)'$) be the leading principal submatrices of $V_k E_s^a(i,k) V_k^*$ (resp., $W_k F_t^b(i,k) W_k^*$) of size $\min \{m_k, n_k\}$. Moreover, set $\phi _{i,k} = \sum _{l=1}^{\min \{m_k,n_k\}} \lambda _{i,k,l} e_l \otimes e_l$, where $e_l$ is the *l*th unit vector in $\mathbb {R}^{\min \{m_k, n_k\}}$. Then we have

$$\begin{aligned} Q_{i,k}(a,b|s,t)&= \mathrm {Tr}\left( E_s^a(i,k) \otimes F_t^b(i,k) \frac{\psi _{i,k} \psi _{i,k}^*}{\Vert \psi _{i,k}\Vert ^2}\right) \\&=\sum _{l,l'=1}^{\min \{m_k, n_k\}} \lambda _{i,k,l} \lambda _{i,k,l'} (v_{i,k,l}^* E_s^a(i,k) v_{i,k,l'})( w_{i,k,l}^* F_t^b(i,k) w_{i,k,l'})\\&= \sum _{l,l'=1}^{\min \{m_k, n_k\}} \lambda _{i,k,l} \lambda _{i,k,l'} (e_l^* E_s^a(i,k)' e_{l'}) ( e_l^* F_t^b(i,k)' e_{l'})\\&= \mathrm {Tr}((E_s^a(i,k)' \otimes F_t^b(i,k)') \phi _{i,k} \phi _{i,k}^*), \end{aligned}$$

which shows $$Q_{i,k}\in C_{q}^{\min \{m_k,n_k\}}(\varGamma )$$.

Combining the convex decompositions $P=\sum _i\lambda _i P_i$ and $P_i= \sum _k \Vert \psi _{i,k}\Vert ^2 Q_{i,k}$, we get the following convex decomposition $P= \sum _{i,k} \lambda _i \Vert \psi _{i,k}\Vert ^2 Q_{i,k}$, from which we obtain

$$\begin{aligned} A_q(P)\le & {} \sum _{i,k} \lambda _i \Vert \psi _{i,k}\Vert ^2 \min \{m_k,n_k\}^2 \le \sum _{i,k} \lambda _i \min \{m_k,n_k\}^2 \\\le & {} \sum _{i,k} \lambda _i m_kn_k= \sum _i \lambda _i d_i. \ \ \ \end{aligned}$$

$$\square $$

We now show that the parameter $A_q(\cdot )$ permits to characterize classical correlations.

#### Proposition 3

For $P\in C_q(\varGamma )$ we have $A_q(P) = 1$ if and only if $P \in C_{loc}(\varGamma )$.

#### Proof

If $P \in C_{loc}(\varGamma )$, then *P* can be written as a convex combination of deterministic correlations (which belong to $C_{q}^1(\varGamma )$), and thus $A_q(P) =1$.

For the reverse implication, assume $A_q(P) = 1$. Then there exist a sequence of convex decompositions $P = \sum _{i \in I^l} \lambda _i^l P_i^{l}$ indexed by $l \in \mathbb {N}$, with $\{P_i^l\} \subseteq C_{q}(\varGamma )$ and $\lim _{l \rightarrow \infty } \sum _{i\in I^l} \lambda _l D_q(P_i^l) = 1$. Decompose the set $I^l$ as the disjoint union $I_-^l \cup I_+^l$, where $D_q(P_i^l)=1$ for $i\in I_-^l$ and $D_q(P_i^l)>1$ for $i\in I_+^l$. Let $\varepsilon > 0$. Then, for all *l* sufficiently large we have

$$\begin{aligned} 1+\sum _{i\in I^l_+}\lambda _i= & {} \left( 1-\sum _{i \in I_+^l} \lambda _i^l\right) + 2 \sum _{i \in I_+^l} \lambda _i^l \le \sum _{i \in I_-^l} \lambda _i^l + \sum _{i \in I_+^l} \lambda _i^l D_q(P_i^l) \\= & {} \sum _{i\in I^l} \lambda _l D_q(P_i^l) \le 1 +\varepsilon , \end{aligned}$$

implying $\smash {\sum _{i \in I_+^l}} \lambda _i^l \le \varepsilon$. This shows that the sequence $\smash {\mu ^l:=\sum _{i\in I^l_-}\lambda _i}$ tends to 1 as $l\rightarrow \infty$. The correlation $\smash {P^l:=\sum _{i\in I^l_-}\lambda ^l_i P^l_i /\mu ^l}$ is a convex combination of deterministic correlations and thus it belongs to $C_{loc}(\varGamma )$. Moreover, $P^l\rightarrow P$ as $l\rightarrow \infty$, which implies $P\in C_{loc}(\varGamma )$. $\square$

As we already observed earlier, when the set $C_q(\varGamma )$ is not closed, the inclusion $C_{q}^d(\varGamma ) \subseteq C_{q}(\varGamma )$ is strict for all *d* (because with a compactness argument one can show that $C_{q}^d(\varGamma )$ is closed), and thus there exists a sequence $\{P_i\}\subseteq C_q(\varGamma )$ with $D_q(P_i)\rightarrow \infty$ as $i\rightarrow \infty$. We show the analogous unboundedness property for the average entanglement dimension $A_q(\cdot )$. For the proof we will use the fact that also the sets $C_{qc}^d(\varGamma )$ are closed for all $d\in \mathbb {N}$.

#### Proposition 4

If $C_{q}(\varGamma )$ is not closed, then there exists a sequence $\{P_i\} \subseteq C_{q}(\varGamma )$ with $A_q(P_i) \rightarrow \infty$.

#### Proof

Assume for contradiction there exists an integer *K* such that $A_q(P) \le K$ for all $P \in C_{q}(\varGamma )$. We will show this results in a uniform upper bound $K'$ on $D_{qc}(P)$, which, in view of (7), implies that $C_{q}(\varGamma )$ is equal to the closed set $C_{qc}^{K'}(\varGamma )$, contradicting the assumption that $C_q(\varGamma )$ is not closed. For this, we will first show that any $P\in C_q(\varGamma )$ belongs to $\mathrm {conv}(C_{qc}^K(\varGamma ))$.

In a first step observe that any $P\in C_{q}(\varGamma ){\setminus } \mathrm {conv}(C_{qc}^K(\varGamma ))$ can be decomposed as

17 $$\begin{aligned} P=\mu _1 R_1+ (1-\mu _1)Q_1, \end{aligned}$$

where $R_1\in C_{q}(\varGamma )$, $Q_1\in \text {conv} (C_{qc}^K(\varGamma ))$, and $0< \mu _1\le K/(K+1)$. Indeed, by assumption and using Proposition 2, $A_{qc}(P)=A_q(P)\le K$, so *P* can be written as a convex combination $P = \sum _{i \in I} \lambda _i P_i$ with $\{P_i\} \subseteq C_{q}(\varGamma )$ and $\sum _{i \in I} \lambda _i D_{qc}(P_i) \le K$. As $P\not \in \mathrm {conv}(C_{qc}^K(\varGamma ))$, the set *J* of indices $i\in I$ with $D_{qc}(P_i)\ge K+1$ is non empty. Then $(K+1) \sum _{i \in J} \lambda _i \le \sum _{i \in J} \lambda _i D_{qc}(P_i) \le K$, and thus $0<\mu _1:=\sum _{i\in J}\lambda _i \le K/(K+1)$. Hence (17) holds after setting $R_1=(\sum _{i\in J}\lambda _i P_i)/\mu _1$ and $Q_1=(\sum _{i\in I\setminus J}\lambda _i P_i)/(1-\mu _1)$.

As $R_1\in C_q(\varGamma )\setminus \mathrm {conv}(C_{qc}^K(\varGamma ))$, we may repeat the same argument for $R_1$. By iterating we obtain for each integer $k\in \mathbb {N}$ a decomposition

$$\begin{aligned} P= & {} \mu _1\mu _2\ldots \mu _k R_k\\&+ \underbrace{(1-\mu _1)Q_1+\mu _1(1-\mu _2)Q_2+\cdots + \mu _1\mu _2\ldots \mu _{k-1}(1-\mu _k)Q_k}_{= (1-\mu _1\mu _2\ldots \mu _k)\hat{Q}_k}, \end{aligned}$$

where $R_k\in C_{q}(\varGamma )$, $\hat{Q}_k\in \text {conv}(C_{qc}^K(\varGamma ))$ and $\mu _1\mu _2\ldots \mu _k\le (K/(K+1))^k$. Then the sequence $\mu _1\mu _2\ldots \mu _{k}$ tends to 0 as $k\rightarrow \infty$. As the entries of $R_k$ lie in [0, 1] we can conclude that $\mu _1\mu _2\ldots \mu _k R_k$ tends to 0 as $k\rightarrow \infty$. Hence the sequence $(\hat{Q}_k)_k$ has a limit $\hat{Q}$ and $P=\hat{Q}$ holds. As all $\hat{Q}_k$ lie in the compact set $\text {conv}(C_{qc}^K(\varGamma ))$, we also have $P\in \text {conv}(C_{qc}^K(\varGamma ))$. So we reach a contradiction, which shows $C_q(\varGamma )\subseteq {\mathrm {conv}}(C_{qc}^K(\varGamma ))$.

The extreme points of the compact convex set $\mathrm {conv}(\smash {C_{qc}^K(\varGamma )})$ lie in $\smash {C_{qc}^K(\varGamma )}$, so, by the Carathéodory theorem, any $P\in \mathrm {conv}(\smash {C_{qc}^K(\varGamma )})$ is a convex combination of *c* elements from $\smash {C_{qc}^K(\varGamma )}$, where $c = |\varGamma |+1-|S||T|$. By using a direct sum construction one can obtain $D_{qc}(P) \le cK$, which shows $K':=cK$ is a uniform upper bound on $D_{qc}(P)$ for all $P\in C_q(\varGamma )$. $\square$

### Setup of the hierarchy

We will now construct a hierarchy of lower bounds on the minimal entanglement dimension, using its formulation via $A_{qc}(\cdot )$. Our approach is based on noncommutative polynomial optimization, thus similar to the approach we used in [16] for bounding matrix factorization ranks.

We first need some notation. Set $\mathbf x=\big \{x^a_s: (a,s)\in A\times S\big \}$ and $\mathbf y=\big \{y^b_t: (b,t)\in B\times T\big \}$, and let $\langle \mathbf x,\mathbf y,z\rangle _r$ be the set of all words in the $n = |S||A|+|T||B| + 1$ symbols $x_s^a$, $y_t^b$, and *z*, having length at most *r*. Moreover, set $\langle \mathbf x, \mathbf y, z \rangle = \langle \mathbf x, \mathbf y, z \rangle _\infty$. We equip $\langle \mathbf x, \mathbf y, z \rangle _r$ with an involution $w \mapsto w^*$ that reverses the order of the symbols in the words and leaves the symbols $x^a_s,y^b_t,z$ invariant; e.g., $(x_s^az)^* = z x_s^a$. Let $\mathbb {R}\langle \mathbf x, \mathbf y, z \rangle _r$ be the vector space of all real linear combinations of the words of length (aka degree) at most *r*. The space $\mathbb {R}\langle \mathbf x, \mathbf y, z \rangle = \mathbb {R}\langle \mathbf x, \mathbf y, z \rangle _\infty$ is the $*$-algebra with Hermitian generators $\{x_s^a\}$, $\{y_t^b\}$, and *z*, and the elements in this algebra are called *noncommutative polynomials* in the variables $\{x^a_s\},\{y^b_t\},z$.

The hierarchy of bounds on $A_{qc}(P)$ is based on the following idea: For any feasible solution to $A_{qc}(P)$, its objective value can be modeled as *L*(1) for a certain tracial linear form *L* on the space of noncommutative polynomials (truncated to degree 2*r*).

Indeed, assume $\{(P_i,\lambda _i)_i\}$ is a feasible solution to the program defining $A_{qc}(P)$ (introduced in Sect. 1.2). That is, $P=\sum _i\lambda _iP_i$ with $\lambda _i\ge 0$, $\sum _i\lambda _i=1$ and $P_i\in C_q(\varGamma )$. Assume $P_i(a,b|s,t) = \mathrm {Tr}\big (X_s^a(i) Y_t^b(i) \psi _i \psi _i^*\big )$ where $\psi \in \mathbb {C}^{d_i}$ and the POVM’s $\{X_s^a(i)\}, \{Y_t^b(i)\} \subset \mathbb {C}^{d_i \times d_i}$ are as in (4), that is, for all $(s,t,a,b) \in \varGamma$ the matrices $X_s^a(i)$ and $Y_t^b(i)$ commute: $[X_s^a(i),Y_t^b(i)] = X_s^a(i) Y_t^b(i) - Y_t^b(i) X_s^a(i) = 0$. For $r\in \mathbb {N}\cup \{\infty \}$, consider the linear functional $L \in \mathbb {R}\langle \mathbf x, \mathbf y, z \rangle _{2r}^*$ defined by

$$\begin{aligned} L(p) = \smash {\sum _i} \lambda _i \, \mathrm {Re}(\mathrm {Tr}(p(\mathbf X(i), \mathbf Y(i), \psi _i \psi _i^*)))\quad \text { for } \quad p\in \mathbb {R}\langle \mathbf x,\mathbf y,z\rangle _{2r}. \end{aligned}$$

Here, for each index *i*, we set

$$\begin{aligned} \mathbf X(i)=(X^a_s(i): (a,s)\in A\times S),\quad \mathbf Y(i)=(Y^b_t(i): (b,t)\in B\times T), \end{aligned}$$

and we replace the variables $x^a_s$, $y^b_t$, *z* by $X^a_s(i)$, $Y^b_t(i)$, and $\psi _i\psi _i^*$, respectively. First note that we have $L(1) = \sum _i \lambda _i d_i$. That is, *L*(1) is equal to the objective value of the feasible solution $\{(P_i, \lambda _i)_i\}$ to $A_{qc}(P)$. Secondly, for all $(s,t,a,b) \in \varGamma$ we have $L(x_s^a y_t^b z) = P(a,b|s,t)$.

We will now identify several computationally tractable properties that this linear functional *L* satisfies. The hierarchy of lower bounds on $A_{qc}(P)$ then consists of optimization problems where we minimize *L*(1) over the set of linear functionals that satisfy these properties.

First note that *L* is *symmetric*, that is, $L(w) = L(w^*)$ for all $w \in \langle \mathbf x, \mathbf y, z\rangle _{2r}$, and *tracial*, that is, $L(ww') = L(w'w)$ for all $w,w' \in \langle \mathbf x, \mathbf y, z\rangle$ with $\deg (ww')\le 2r$.

Next, for all $p \in \mathbb {R}\langle \mathbf x, \mathbf y, z\rangle _{r-1}$ we have

$$\begin{aligned} L(p^*x_s^ap)= & {} \sum _i \lambda _i \, \mathrm {Re}(\mathrm {Tr}(C(i)^* X_s^a(i) C(i)) \ge 0, \end{aligned}$$

where $C(i) = p(\mathbf X(i), \mathbf Y(i), \psi _i \psi _i^*)$, as $C(i)^* X_s^a(i) C(i)$ is positive semidefinite since $X^a_s(i)$ is positive semidefinite. In the same way one can check that $L(p^*y_t^bp) \ge 0$ and $L(p^*z p) \ge 0$. That is, if we set

$$\begin{aligned} \mathscr {G} = \big \{x_s^a : s \in S, \, a \in A\big \} \cup \big \{y_t^b : t \in T, \, b \in B\big \}\cup \{z\}, \end{aligned}$$

then *L* is nonnegative (denoted as $L \ge 0$) on the *truncated quadratic module*

18 $$\begin{aligned} {\mathscr {M}}_{2r}(\mathscr {G})=\mathrm {cone}\Big \{p^*g p: p\in \mathbb {R}\langle \mathbf x, \mathbf y, z\rangle , \ g\in \mathscr {G}\cup \{1\},\ \deg (p^*g p)\le 2r\Big \}. \end{aligned}$$

Similarly, setting

$$\begin{aligned} \mathscr {H}= & {} \big \{z - z^2\big \} \cup \big \{1 - \sum _{a\in A} x_s^a : s \in S\big \} \\&\cup \big \{1 - \sum _{b\in B} y_t^b : t \in T\big \}\cup \big \{[x_s^a, y_t^b] : (s,t,a,b) \in \varGamma \big \}, \end{aligned}$$

we have $L = 0$ on the *truncated ideal*

19 $$\begin{aligned} \mathscr {I}_{2r}(\mathscr {H}) = \Big \{ph : p\in \mathbb {R}\langle \mathbf x, \mathbf y, z\rangle , \ h\in \mathscr {H},\ \deg (ph)\le 2r\Big \}. \end{aligned}$$

Moreover, we have $L(z) = \sum _i\lambda _i \mathrm {Re}({{\mathrm{Tr}}}(\psi _i\psi _i^*))=1$. In addition, for any matrices $U,V \in \mathbb {C}^{d_i \times d_i}$ we have

$$\begin{aligned} \psi _i \psi _i^* U \psi _i \psi _i^* V \psi _i \psi _i^* = \psi _i \psi _i^* V \psi _i \psi _i^* U \psi _i \psi _i^*, \end{aligned}$$

and therefore, in particular,

$$\begin{aligned} L(w z u z v z) = L(wz v z u z) \quad \text {for all} \quad u,v,w \in \langle \mathbf x, \mathbf y,z\rangle \quad \text {with} \quad \deg (w z u z v z ) \le 2r. \end{aligned}$$

That is, we have $L = 0$ on $\mathscr {I}_{2r}(\mathscr {R}_r)$, where

$$\begin{aligned} \mathscr {R}_r = \big \{z u z v z - z v z u z : u,v \in u,v \in \langle \mathbf x, \mathbf y,z\rangle \text { with } \deg (z u z v z) \le 2r\big \}. \end{aligned}$$

We get the idea of adding these last constraints from [37], where this is used to study the mutually unbiased bases problem.

We call ${\mathscr {M}}(\mathscr {G}) = {\mathscr {M}}_\infty (\mathscr {G})$ the quadratic module generated by $\mathscr {G}$, and we call ${\mathscr {I}}(\mathscr {H} \cup \mathscr {R}_\infty ) = {\mathscr {I}}_\infty (\mathscr {H} \cup \mathscr {R}_\infty )$ the ideal generated by $\mathscr {H}\cup \mathscr {R}_\infty$.

For $r \in \mathbb {N}\cup \{\infty \}$ we can now define the parameter:

$$\begin{aligned} {\xi _{r}^{\mathrm {q}}}(P) = \mathrm {min} \Big \{ L(1) : \;&L \in \mathbb {R}\langle \mathbf x, \mathbf y, z \rangle _{2r}^* \text { tracial and symmetric},\\&L(z) = 1, \, L(x_s^a y_t^b z) = P(a,b|s,t) \text { for all } (a,b,s,t)\in \varGamma ,\\&L \ge 0 \text { on } \mathscr {M}_{2r}(\mathscr {G}),\, L = 0 \text { on } \mathscr {I}_{2r}(\mathscr {H} \cup \mathscr {R}_r)\Big \}. \end{aligned}$$

Note that for order $r=1$ we get the trivial bound ${\xi _{1}^{\mathrm {q}}}(P)=1$.

For each finite $r\in \mathbb {N}$ the parameter ${\xi _{r}^{\mathrm {q}}}(P)$ can be computed by semidefinite programming. Indeed, the condition $L\ge 0$ on $\mathscr {M}_{2r}(\mathscr {G})$ means that $L(p^*gp)\ge 0$ for all $g \in \mathscr {G} \cup \{1\}$ and all polynomials $p\in \mathbb {R}\langle \mathbf x,\mathbf y,z\rangle$ with degree at most ${r- \lceil \deg (g)/2\rceil }$. This is equivalent to requiring that the matrices $(L(w^*gw'))$, indexed by all words $w,w'$ with degree at most ${r- \lceil \deg (g)/2\rceil }$, are positive semidefinite. To see this, write $p = \sum _{w} p_w w$ and let $\hat{p}=(p_w)$ denote the vector of coefficients, then $L(p^*gp) \ge 0$ is equivalent to $\hat{p}^\mathsf{T}(L(w^*g w')) \hat{p} \ge 0$. When $g=1$, the matrix $(L(w^*w'))$ is indexed by the words of degree at most *r*, it is called the *moment matrix* of *L* and denoted by $M_r(L)$ (or *M*(*L*) when $r = \infty$). The entries of the matrices $(L(w^*g w'))$ are linear combinations of the entries of $M_r(L)$, and the constraint $L=0$ on ${\mathscr {I}}_{2r}(\mathscr {H} \cup \mathscr {R}_r)$ can be written as a set of linear constraints on the entries of $M_r(L)$. It follows that for finite $r \in \mathbb {N}$, the parameter ${\xi _{r}^{\mathrm {q}}}(P)$ is indeed computable by a semidefinite program.

Additionally, we define the parameter ${\xi _{*}^{\mathrm {q}}}(P)$ by adding to the definition of ${\xi _{\infty }^{\mathrm {q}}}(P)$ the constraint ${{\mathrm{rank}}}(M(L)) < \infty$. By construction this gives a hierarchy of lower bounds for $A_{qc}(P)$:

$$\begin{aligned} {\xi _{1}^{\mathrm {q}}}(P)\le \ldots \le {\xi _{r}^{\mathrm {q}}}(P) \le {\xi _{\infty }^{\mathrm {q}}}(P)\le {\xi _{*}^{\mathrm {q}}}(P) \le A_{qc}(P). \end{aligned}$$

Indeed, if $L\in \mathbb {R}\langle \mathbf x, \mathbf y, z \rangle _{2r}^*$ is feasible for $\xi _r^q(P)$ then its restriction to $\mathbb {R}\langle \mathbf x, \mathbf y, z \rangle _{2r-2}^*$ is feasible for $\xi ^q_{r-1}(P)$, which implies $\xi ^q_{r-1}(P) \le L(1)$ and thus $\xi ^q_{r-1}(P)\le \xi ^q_r(P)$.

### Background on positive tracial linear forms

Before we show the convergence results for the hierarchy $\{\xi ^q_r(P)\}$ we give some background on positive tracial linear forms, which we will use again in Sect. 4. We state these results using the variables $x_1,\ldots ,x_n$, where we use the notation $\langle \mathbf x\rangle = \langle x_1,\ldots ,x_n\rangle$. The results stated below do not always appear in this way in the sources cited; we follow the presentation of [16], where full proofs for all these results are also provided.

First we need a few more definitions. A polynomial $p \in \mathbb {R}\langle \mathbf{x}\rangle$ is called symmetric if $p^*=p$, and we denote the set of symmetric polynomials by $\mathrm {Sym}\, \mathbb {R}\langle \mathbf x\rangle$. Given $\mathscr {G} \subseteq \mathrm {Sym} \, \mathbb {R}\langle \mathbf x\rangle$ and $\mathscr {H} \subseteq \mathbb {R}\langle \mathbf{x}\rangle$, the set $\mathscr {M}(\mathscr {G}) + \mathscr {I}(\mathscr {H})$ is called *Archimedean* if it contains the polynomial $R-\sum _{i=1}^n x_i^2$ for some $R > 0$.

Recall that for our purposes a $C^*$-algebra $\mathscr {A}$ can be defined as a norm closed $*$-subalgebra of the space ${\mathscr {B}}(H)$ of bounded operators on a complex Hilbert space *H*. We say that a $C^*$-algebra ${\mathscr {A}}$ is *unital* if it contains the identity operator (denoted 1). An element $a \in {\mathscr {A}}$ is called *positive* if $a = b^*b$ for some $b \in {\mathscr {A}}$. A linear form $\tau$ on a unital $C^*$-algebra ${\mathscr {A}}$ is said to be a *state* if $\tau (1)=1$ and $\tau$ is positive; that is, $\tau (a) \ge 0$ for all positive elements $a \in {\mathscr {A}}$. We say that a state $\tau$ is tracial if $\tau (ab)=\tau (ba)$ for all $a,b\in {\mathscr {A}}$. See, for example, [4] for more information on $C^*$-algebras.

The first result relates positive tracial linear forms to $C^*$-algebras; see [37] for the noncommutative (eigenvalue) setting and [8] for the tracial setting.

#### Theorem 2

Let $\mathscr {G} \subseteq \mathrm {Sym}\,\mathbb {R}\langle \mathbf{x}\rangle$ and $\mathscr {H} \subseteq \mathbb {R}\langle \mathbf{x}\rangle$ and assume that ${\mathscr {M}}(\mathscr {G})+ \mathscr {I}(\mathscr {H})$ is Archimedean. For a linear form $L\in \mathbb {R}\langle \mathbf{x}\rangle ^*$, the following are equivalent:

1. *L* is symmetric, tracial, nonnegative on ${\mathscr {M}}(\mathscr {G})$, zero on $\mathscr {I}(\mathscr {H})$, and $L(1) = 1$;
2. there is a unital $C^*$-algebra $\mathscr {A}$ with tracial state $\tau$ and $\mathbf{X} \in \mathscr {A}^n$ such that $g(\mathbf X)$ is positive in $\mathscr {A}$ for all $g \in \mathscr {G}$, and $h(\mathbf X) = 0$ for all $h \in \mathscr {H}$, with
  20 $$\begin{aligned} L(p)=\tau (p(\mathbf{X})) \quad \text {for all} \quad p\in \mathbb {R}\langle \mathbf{x}\rangle . \end{aligned}$$

The following can be seen as the finite dimensional analogue of the above result. The proof of the unconstrained case ($\mathscr {G} = \mathscr {H} = \emptyset$) can be found in [7], and for the constrained case in [8].

Given a linear form $L \in \mathbb {R}\langle \mathbf{x}\rangle ^*$, recall that its moment matrix *M*(*L*) is given by $M(L)_{u,v} = L(u^*v)$ for $u,v \in \langle \mathbf{x}\rangle$. Recall also that *L* is called a *normalized trace evaluation* if there exists a tuple $(X_1,\ldots ,X_n)$ of $d\times d$ Hermitian matrices (for some $d\in \mathbb {N}$) such that $L(p)=\text {Tr}(p(X_1,\ldots ,X_n))/d$ for all $p\in \mathbb {R}\langle \mathbf{x}\rangle$.

#### Theorem 3

Let $\mathscr {G} \subseteq \mathrm {Sym}\,\mathbb {R}\langle \mathbf{x}\rangle$ and $\mathscr {H} \subseteq \mathbb {R}\langle \mathbf{x}\rangle$. For $L\in \mathbb {R}\langle \mathbf{x}\rangle ^*$, the following are equivalent:

1. *L* is a symmetric, tracial, linear form with $L(1) =1$ that is nonnegative on ${\mathscr {M}}(\mathscr {G})$, zero on $\mathscr {I}(\mathscr {H})$, and has $\mathrm {rank}(M(L)) < \infty$;
2. there is a finite dimensional $C^*$-algebra $\mathscr {A}$ with a tracial state $\tau$ and $\mathbf{X} \in \mathscr {A}^n$ satisfying (20), with $g(\mathbf X)$ positive in $\mathscr {A}$ for all $g \in \mathscr {G}$ and $h(\mathbf X) = 0$ for all $h \in \mathscr {H}$;
3. *L* is a convex combination of normalized trace evaluations at tuples $\mathbf{X}$ of Hermitian matrices that satisfy $g(\mathbf X) \succeq 0$ for all $g \in \mathscr {G}$ and $h(\mathbf X) = 0$ for all $h \in \mathscr {H}$.

Given an integer $r\in \mathbb {N}$ a (truncated) linear functional $L \in \mathbb {R}\langle \mathbf{x}\rangle _{2r}$ is called $\delta$*-flat* if the principal submatrix $M_{r-\delta }(L)$ of $M_r(L)$ indexed by monomials up to degree $r-\delta$ has the same rank as $M_r(L)$, i.e.,

21 $$\begin{aligned} {{\mathrm{rank}}}(M_r(L))={{\mathrm{rank}}}(M_{r-\delta }(L)). \end{aligned}$$

One says *L* is *flat* if it is $\delta$-flat for some $\delta \ge 1$. The following result claims that any flat linear functional on a truncated polynomial space can be extended to a linear functional *L* on the full algebra of polynomials. It is due to Curto and Fialkow [11] in the commutative case and extensions to the noncommutative case can be found in [48] (for eigenvalue optimization) and [7, 22] (for trace optimization).

#### Theorem 4

Let $1 \le \delta \le r < \infty$, $\mathscr {G} \subseteq \mathrm {Sym}\,\mathbb {R}\langle \mathbf{x}\rangle _{2\delta }$, and $\mathscr {H} \subseteq \mathbb {R}\langle \mathbf{x}\rangle _{2\delta }$. If $L\in \mathbb {R}\langle \mathbf{x}\rangle _{2r}^*$ is symmetric, tracial, $\delta$-flat, nonnegative on ${\mathscr {M}}_{2r}(\mathscr {G})$, and zero on $\mathscr {I}_{2r}(\mathscr {H})$, then *L* extends to a symmetric, tracial, linear form on $\mathbb {R}\langle \mathbf{x}\rangle$ that is nonnegative on $\mathscr {M}(\mathscr {G})$, zero on $\mathscr {I}(\mathscr {H})$, and whose moment matrix *M*(*L*) has finite rank.

The following technical lemma, based on the Banach-Alaoglu theorem, is a well-known tool to show asymptotic convergence results in polynomial optimization.

#### Lemma 1

Let $\mathscr {G} \subseteq \mathrm {Sym}\, \mathbb {R}\langle \mathbf{x}\rangle$, $\mathscr {H} \subseteq \mathbb {R}\langle \mathbf{x}\rangle$, and assume that for some $d \in \mathbb {N}$ and $R>0$ we have $R-(x_1^2 + \cdots + x_n^2) \in {\mathscr {M}}_{2d}(\mathscr {G})+\mathscr {I}_{2d}(\mathscr {H})$. For $r\in \mathbb {N}$ assume $L_r \in \smash {\mathbb {R}\langle \mathbf x\rangle _{2r}^*}$ is tracial, nonnegative on $\mathscr {M}_{2r}(\mathscr {G})$ and zero on ${\mathscr {I}}_{2r}(\mathscr {H})$. Then $\smash {|L_r(w)|\le R^{|w|/2} L_r(1)}$ for all $w\in \langle \mathbf{x}\rangle _{2r-2d+2}$. In addition, if $\sup _r \, L_r(1) < \infty$, then $\smash {\{L_r\}}_r$ has a pointwise converging subsequence in $\smash {\mathbb {R}\langle \mathbf x\rangle ^*}$.

### Convergence results

We first show that the parameter $\xi ^q_*(P)$ coincides with the average entanglement dimension $A_q(P)$ and then we consider convergence properties of the bounds ${\xi _{r}^{\mathrm {q}}}(P)$ to the parameters ${\xi _{\infty }^{\mathrm {q}}}(P)$ and ${\xi _{*}^{\mathrm {q}}}(P)$.

#### Proposition 5

For any $P\in C_{q}(\varGamma )$ we have ${\xi _{*}^{\mathrm {q}}}(P) = A_{qc}(P)$.

#### Proof

We already know ${\xi _{*}^{\mathrm {q}}}(P) \le A_{qc}(P)$. To show ${\xi _{*}^{\mathrm {q}}}(P) \ge A_{qc}(P)$ we let *L* be feasible for ${\xi _{*}^{\mathrm {q}}}(P)$, so that $L \ge 0$ on $\mathscr {M}(\mathscr {G})$, $L = 0$ on $\mathscr {I}(\mathscr {H} \cup \mathscr {R}_\infty )$ and ${{\mathrm{rank}}}(M(L))<\infty$. We apply Theorem 3 to the scaled linear form *L* / *L*(1) (note that $L(1)>0$ since $L(z)=1$): there exist finitely many scalars $\lambda _i \ge 0$ with $\sum _i\lambda _i=L(1)$, Hermitian matrix tuples $\mathbf {X}(i) = (X^a_s(i))_{a,s}$ and $\mathbf {Y}(i)= (Y^b_t(i))_{b,t}$, and Hermitian matrices $Z_i$, so that

22 $$\begin{aligned} g(\mathbf {X}(i), \mathbf {Y}(i), Z_i) \succeq 0\ \text { for all } g \in \mathscr {G}, \ \ h(\mathbf {X}(i), \mathbf {Y}(i), Z_i) = 0\ \text { for all } h \in \mathscr {H} \cup \mathscr {R}_\infty , \end{aligned}$$

and

23 $$\begin{aligned} L(p) = \smash {\sum _i} \lambda _i\, \mathrm {Tr}(p(\mathbf {X}(i), \mathbf {Y}(i), Z_i)) \quad \text {for all} \quad p \in \mathbb {R}\langle \mathbf x, \mathbf y, z\rangle . \end{aligned}$$

By Artin–Wedderburn theory (Theorem 1) we know that for each *i* there is a unitary matrix $V_i$ such that $V_i \mathbb {C}\langle \mathbf X(i), \mathbf Y(i), Z_i\rangle V_i^* = \bigoplus _k \mathbb {C}^{d_k \times d_k} \otimes I_{m_k}$. Hence, after applying this further block diagonalization we may assume that in the decomposition (23), for each *i*, $\mathbb {C}\langle \mathbf X(i), \mathbf Y(i), Z_i\rangle$ is a full matrix algebra $\mathbb {C}^{d_{ i }\times d_{ i}}$.

Since $$h(\mathbf {X}(i), \mathbf {Y}(i), Z_i) = 0$$ for all $$h \in R_\infty \cup \{z-z^2\}$$, $$Z_i$$ is a projector and the commutator $$\big [Z_i u Z_i, Z_i v Z_i\big ]$$ vanishes for all $$u, v \in \langle \mathbf {X}(i), \mathbf {Y}(i), Z_i\rangle $$ and hence for all $$u,v \in \mathbb {C}\langle \mathbf {X}(i), \mathbf {Y}(i), Z_i\rangle $$. This means that $$ [Z_i T_1 Z_i, Z_i T_2 Z_i] = 0$$ for all $$T_1,T_2 \in \mathbb {C}^{d_i \times d_i}. $$ As $$Z_i$$ is a projector, there exists a unitary matrix $$U_i$$ such that $$ U_iZ_iU_i^* = \mathrm {Diag}(1,\ldots ,1,0,\ldots ,0). $$ The above then implies that for all $$T_1$$ and $$T_2$$, the leading principal submatrices of size $$\mathrm {rank}(Z_i)$$ of $$U_iT_1U_i^*$$ and $$U_iT_2U_i^*$$ commute. This implies $$\mathrm {rank}(Z_{i}) \le 1$$ and thus $${{\mathrm{Tr}}}(Z_i) \in \{0,1\}$$. Let *I* be the set of indices with $$\mathrm {Tr}(Z_i) = 1$$. Then we have $$\sum _{i \in I} \lambda _i = \sum _i \lambda _i \, \mathrm {Tr}(Z_i) = L(z) = 1$$.

For each $i\in I$ define $P_i=\smash {(\mathrm {Tr}( X_s^a(i) Y_t^b(i) Z_i))}$, which is a quantum correlation in $C_{qc}^{d_i}(\varGamma )$ because ${{\mathrm{Tr}}}(Z_i)=1$, and $X^a_s,Y^b_t\succeq 0$ with $\sum _a X^a_s(i)=\sum _b Y^b_t(i)=I$ and $[X^a_s(i),Y^b_t(i)]=0$ in view of (22). Using (23) we obtain $P=\sum _{i \in I} \lambda _i P_i$. Hence, $(P_i,\lambda _i)_{i\in I}$ forms a feasible solution to $A_{qc}(P)$ with objective value $\sum _{i\in I} \lambda _iD_{qc}(P_i)\le \sum _{i \in I} \lambda _i d_i \le \sum _{i} \lambda _i d_i = L(1).$ $\square$

The problem ${\xi _{r}^{\mathrm {q}}}(P)$ differs in two ways from a standard tracial optimization problem. First it does not have the normalization $L(1) = 1$ (and instead it minimizes *L*(1)), and second it has ideal constraints $L = 0$ on $\mathscr {I}_{2r}(\mathscr {R}_r)$ where $\mathscr {R}_r$ depends on the relaxation order *r*. Nevertheless we can show that asymptotic convergence still holds.

#### Proposition 6

For any $P\in C_{q}(\varGamma )$ we have ${\xi _{r}^{\mathrm {q}}}(P)\rightarrow {\xi _{\infty }^{\mathrm {q}}}(P)$ as $r\rightarrow \infty$.

#### Proof

First observe that $1-z^2$, $1-(x_s^a)^2$, $1-(y_t^b)^2 \in {\mathscr {M}}_4(\mathscr {G} \cup \mathscr {H}_0)$, where $\mathscr {H}_0$ contains the symmetric polynomials in $\mathscr {H}$; i.e., omitting the commutators $[x^a_s, y^b_t]$. Indeed, we have $1-z^2= (1-z)^2+2(z-z^2)$ and

$$\begin{aligned} 1-(x_s^a)^2= (1-x_s^a)^2+2(1-x^a_s)x^a_s(1-x^a_s)+ 2x^a_s\left( \left( 1-\sum _{a'} x^{a'}_s\right) + \sum _{a'\ne a}x^{a'}_s\right) x^a_s, \end{aligned}$$

and the same for $y_t^b$. Hence $R-z^2-\sum _{a,s}(x^a_s)^2-\sum _{b,t}(y^b_t)^2\in \mathscr {M}_4(\mathscr {G}\cup \mathscr {H}_0)$ for some $R>0$. Fix $\varepsilon >0$ and for each $r\in \mathbb {N}$ let $L_r$ be feasible for ${\xi _{r}^{\mathrm {q}}}(P)$ with value $L_r(1)\le {\xi _{r}^{\mathrm {q}}}(P)+\varepsilon$. As $L_r$ is tracial and zero on $\mathscr {I}_{2r}(\mathscr {H}_0)$, it follows (using the identity $p^* gp = pp^*g + [p^*g,p]$) that $L=0$ on $\mathscr {M}_{2r}(\mathscr {H}_0)$. Hence, $L_r\ge 0$ on $\mathscr {M}_{2r}(\mathscr {G}\cup \mathscr {H}_0)$. Since $\sup _rL_r(1)\le A_q(P)+\varepsilon$, we can apply Lemma 1 and conclude that $\{L_r\}_r$ has a converging subsequence; denote its limit by $L_\varepsilon \in \mathbb {R}\langle \mathbf{x}\rangle ^*$. One can verify that $L_\varepsilon$ is feasible for ${\xi _{\infty }^{\mathrm {q}}}(P)$, and ${\xi _{\infty }^{\mathrm {q}}}(P)\le L_\varepsilon (1)\le \lim _{r\rightarrow \infty } {\xi _{r}^{\mathrm {q}}}(P) +\varepsilon \le {\xi _{\infty }^{\mathrm {q}}}(P)+\varepsilon .$ Letting $\varepsilon \rightarrow 0$ we obtain that ${\xi _{\infty }^{\mathrm {q}}}(P)=\lim _{r\rightarrow \infty }{\xi _{r}^{\mathrm {q}}}(P)$. $\square$

Next we show that finite convergence holds under a certain flatness condition: if ${\xi _{r}^{\mathrm {q}}}(P)$ admits a $\delta$-flat optimal solution with $\delta =\lceil r/3 \rceil +1$, then ${\xi _{r}^{\mathrm {q}}}(P) = {\xi _{*}^{\mathrm {q}}}(P)$. This result is a variation of the flat extension result from Theorem 4, where $\delta$ now depends on the order *r* because the ideal constraints in ${\xi _{r}^{\mathrm {q}}}(P)$ depend on *r*.

#### Proposition 7

If ${\xi _{r}^{\mathrm {q}}}(P)$ admits a $(\lceil r/3 \rceil +1)$-flat optimal solution, then we have ${\xi _{r}^{\mathrm {q}}}(P) = {\xi _{*}^{\mathrm {q}}}(P)$.

#### Proof

Let $\delta = \lceil r/3 \rceil +1$ and let *L* be a $\delta$-flat optimal solution to ${\xi _{r}^{\mathrm {q}}}(P)$, i.e., such that ${{\mathrm{rank}}}(M_r(L))={{\mathrm{rank}}}(M_{r-\delta }(L))$. We have to show ${\xi _{r}^{\mathrm {q}}}(P) \ge {\xi _{*}^{\mathrm {q}}}(P)$, which we do by constructing a feasible solution $\hat{L}$ to ${\xi _{*}^{\mathrm {q}}}(P)$ with the same objective value $\hat{L}(1)=L(1)$. In the proof of Theorem 4 (see [16, Thm. 2.3], and also [22, Prop. 6.1] for the original proof of this theorem), the linear form *L* is extended to a tracial symmetric linear form $\smash {\hat{L}}$ on $\mathbb {R}\langle \mathbf x, \mathbf y, z \rangle$ that is nonnegative on $\mathscr {M}(\mathscr {G})$, zero on $\mathscr {I}(\mathscr {H})$, with $\mathrm {rank}(M(\hat{L})) < \infty$. To do this a subset *W* of $\langle \mathbf x, \mathbf y, z \rangle _{r-\delta }$ is found such that we have the vector space direct sum $\mathbb {R}\langle \mathbf x, \mathbf y, z \rangle = \mathrm {span}(W) \oplus \mathscr {I}(N_r(L)),$ where $N_r(L)$ is the vector space

$$\begin{aligned} N_r(L) = \big \{ p \in \mathbb {R}\langle \mathbf x, \mathbf y, z \rangle _r : L(qp) = 0 \text { for all } q \in \mathbb {R}\langle \mathbf x, \mathbf y, z \rangle _r \big \}. \end{aligned}$$

It is moreover shown that $\mathscr {I}(N_r(L)) \subseteq N(\hat{L})$. For $p \in \mathbb {R}\langle \mathbf x, \mathbf y, z \rangle$ we denote by $r_p$ the unique element in $\mathrm {span}(W)$ such that $p - r_p \in \mathscr {I}(N_r(L))$.

We show that $\hat{L}$ is zero on $\mathscr {I}(\mathscr {R}_\infty )$. Fix $u,v, w \in \mathbb {R}\langle \mathbf x, \mathbf y, z\rangle$. Then we have

$$\begin{aligned} \hat{L}(w(z u z v z - z v z u z)) = \hat{L}(w z uz v z ) -\hat{L}(wz v z u z ). \end{aligned}$$

Since $\hat{L}$ is tracial and $u -r_u, v - r_v, w-r_w \in \mathscr {I}(N_r(L)) \subseteq N(\hat{L})$, we have

$$\begin{aligned} \hat{L}(wz u z vz) = \hat{L}(r_w z r_u z r_v z) \quad \text {and} \quad \hat{L}(wz v z uz) = \hat{L}(r_w z r_v z r_u z). \end{aligned}$$

Since $\mathrm {deg}(r_u z r_v z r_wz) = \mathrm {deg}(r_v z r_u z r_wz) \le 3+3(r-\delta )\le 2r$ we have

$$\begin{aligned} \hat{L}(r_w z r_u z r_v z) = L(r_w z r_u z r_v z) \quad \text {and} \quad \hat{L}(r_w z r_v z r_u z) = L(r_w z r_v z r_u z). \end{aligned}$$

So $L =0$ on $\mathscr {I}_{2r}(\mathscr {R}_r)$ implies $\hat{L}=0$ on $\mathscr {I}(\mathscr {R}_\infty )$.

Since $\hat{L}$ extends *L* we have $\hat{L}(z) = L(z) = 1$ and $\hat{L}(x_s^ay_t^bz) = L(x_s^ay_t^bz) = P(a,b|s,t)$ for all *a*, *b*, *s*, *t*. So, $\hat{L}$ is feasible for ${\xi _{*}^{\mathrm {q}}}(P)$ and has the same objective value $\hat{L}(1) = L(1)$. $\square$

## Bounding quantum graph parameters

We investigate the quantum graph parameters $\alpha _q(G)$, $\gamma _q(G)$, $\alpha _{qc}(G)$, and $\chi _{qc}(G)$, which are quantum analogues of the classical graph parameters $\alpha (G)$ and $\chi (G)$. They were introduced earlier in Sect. 1.3 in terms of nonlocal games and synchronous quantum correlations (in the tensor and commuting models). As we will see below, they can be reformulated in terms of the existence of positive semidefinite matrices with arbitrary size (or operators) satisfying a system of equations corresponding to the natural integer linear programming formulation of $\alpha (G)$ and $\chi (G)$. This opens the way to using techniques from noncommutative polynomial optimization for designing hierarchies of bounds for the quantum graph parameters. We present these approaches and compare them with known hierarchies for the classical graph parameters.

### Hierarchies $\gamma _r^\mathrm {col}(G)$ and $\gamma _r^\mathrm {stab}(G)$ based on synchronous correlations

In Sect. 1.3 we introduced quantum chromatic numbers (Definition 1) and quantum stability numbers (Definition 2) in terms of synchronous quantum correlations satisfying certain linear constraints. We first give (known) reformulations in terms of $C^*$-algebras, and then we reformulate those in terms of tracial optimization, which leads to the hierarchies $\gamma _r^\mathrm {col}(G)$ and $\gamma _r^\mathrm {stab}(G)$.

The following result from [46] allows us to write a synchronous quantum correlation in terms of $C^*$-algebras admitting a tracial state.

#### Theorem 5

([46]) Let $\varGamma = A^2 \times S^2$ and $P\in \mathbb {R}^\varGamma$. We have $P \in C_{qc,s}(\varGamma )$ (resp., $P \in C_{q,s}(\varGamma )$) if and only if there exists a unital (resp., finite dimensional) $C^*$-algebra ${\mathscr {A}}$ with a faithful tracial state $\tau$ and a set of projectors $\{X_s^a: s \in S, a \in A\}\, \textit{in}\, {\mathscr {A}}$ satisfying $\sum _{a \in A} X_s^a = 1$ for all $s \in S$ and $P(a,b|s,t) = \tau (X_s^a X_t^b)$ for all $s,t \in S$ and $a,b \in A$.

Here we add the condition that $\tau$ is faithful, that is, $\tau (X^*X) = 0$ implies $X=0$, since it follows from the GNS construction in the proof of [46]. This means that

$$\begin{aligned} 0 = P(a,b|s,t) = \tau (X_s^a X_t^b) = \tau \left( \left( X_s^a\right) ^2 \big (X_t^b\big )^2\right) = \tau \left( \left( X_s^a X_t^b\right) ^* X_s^a X_t^b\right) \end{aligned}$$

implies $X_s^a X_t^b = 0$. It follows from Definition 1 and the above that $\chi _{qc}(G)$ is equal to the smallest $k \in \mathbb {N}$ for which there exists a $C^*$-algebra ${\mathscr {A}}$, a tracial state $\tau$ on ${\mathscr {A}}$, and a family of projectors $\{X_i^c: i \in V, c \in [k]\}\subseteq {\mathscr {A}}$ satisfying

24 $$\begin{aligned}&\sum _{c \in [k]} X_i^c -1 = 0 \quad \text {for all} \quad i \in V, \end{aligned}$$

25 $$\begin{aligned}&X_i^c X_j^{c'} = 0 \quad \text {if} \quad (c \ne c' \text { and }i=j ) \quad \text {or} \quad (c=c' \text { and } \{i,j\}\in E). \end{aligned}$$

The quantum chromatic number $\chi _q(G)$ is equal to the smallest $k \in \mathbb {N}$ for which there exists a finite dimensional $C^*$-algebra ${\mathscr {A}}$ with the above properties.

Analogously, $\alpha _{qc}(G)$ is equal to the largest $k \in \mathbb {N}$ for which there is a $C^*$-algebra ${\mathscr {A}}$, a tracial state $\tau$ on ${\mathscr {A}}$, and a set of projectors $\{X_c^i: c \in [k], i \in V\}\subseteq {\mathscr {A}}$ satisfying

26 $$\begin{aligned}&\sum _{i \in V} X_c^i -1 = 0 \quad \text {for all} \quad c \in [k], \end{aligned}$$

27 $$\begin{aligned}&X_c^i X_{c'}^j = 0 \quad \text {if } (i \ne j \text { and } c=c') \quad \text {or} \quad ((i=j \text { or } \{i,j\}\in E) \text { and } c\ne c'),\nonumber \\ \end{aligned}$$

and $\alpha _q(G)$ is equal to the largest $k \in \mathbb {N}$ for which $\mathscr {A}$ can be taken finite dimensional.

These reformulations of $\chi _q(G), \chi _{qc}(G), \alpha _q(G)$ and $\alpha _{qc}(G)$ also follow from [41, Thm. 4.7], where general quantum graph homomorphisms are considered; the formulations of $\chi _q(G)$ and $\chi _{qc}(G)$ are also made explicit in [41, Thm. 4.12].

#### Remark 1

The above definition for the parameters $\alpha _q(G)$ and $\chi _q(G)$ (tensor model) can be simplified. Indeed, instead of asking for projectors $\{X^c_i\}$ living in a finite dimensional $C^*$-algebra equipped with a tracial state and satisfying the constraints (24)–(25) or (26)–(27), one may ask for such projectors that are *matrices* of unspecified (but finite) size (as in [9, 32, 52]). This can be seen in the following two ways.

A first possibility is to apply Artin–Wedderburn theory, which tells us that any finite dimensional $C^*$-algebra is isomorphic to a matrix algebra.

An alternative, more elementary way is to use the link presented in Sect. 2 between synchronous quantum correlations and completely positive semidefinite matrices. Indeed, as we have seen there, having a synchronous quantum correlation $P=(P(c,c'|i,j))\in \mathbb {R}^{V^2\times [k]^2}$ certifying $\chi _q(G)\le k$ is equivalent to having a set of positive semidefinite matrices $\{X^c_i\}$ satisfying the constraints (24)–(25). Here we use the basic fact that since $X^c_i,X^{c'}_j\succeq 0$, we have $P(c,c'|i,j)=\text {Tr}(X^c_iX^{c'}_j)=0$ if and only if $\smash {X^c_iX^{c'}_j}=0$. Next, observe that the constraints (24)–(25) imply that the matrices $X_i^c$ are projectors. Indeed, for every $i,c'$, by multiplying (24) by $X_i^{c'}$ and using (25) we obtain $(X_i^{c'})^2 = X_i^{c'}$. The analogous result holds of course for the quantum stability number $\alpha _q(G)$.

Finally, note that restricting to scalar solutions ($1 \times 1$ matrices) in these feasibility problems recovers the classical graph parameters $\chi (G)$ and $\alpha (G)$.

We now reinterpret the above formulations in terms of tracial optimization. Given a graph $G = (V,E)$, let $i \simeq j$ denote $\{i,j\} \in E$ or $i =j$. For $k \in \mathbb {N}$, let $\mathscr {H}_{G,k}^\mathrm{col}$ and $\mathscr {H}_{G,k}^\mathrm{stab}$ denote the sets of polynomials corresponding to equations (24)–(25) and (26)–(27):

$$\begin{aligned} \mathscr {H}^\mathrm{col}_{G,k}&=\left\{ 1-\sum _{c\in [k]}x^c_i : i\in V\right\} \\&\qquad \cup \left\{ x^c_i x^{c'}_j :(c \ne c' \text { and }i=j ) \text { or }(c=c' \text { and } \{i,j\}\in E) \right\} , \\ \mathscr {H}^\mathrm{stab}_{G,k}&= \left\{ 1-\sum _{i\in V}x^i_c : c\in [k]\right\} \cup \left\{ x^i_cx^j_{c'} : (i \ne j \text { and } c=c') \text { or } (i \simeq j \text { and } c\ne c')\right\} . \end{aligned}$$

We have

$$\begin{aligned} 1-\left( x_i^c\right) ^2 \in {\mathscr {M}}_2(\emptyset ) + {\mathscr {I}}_2\left( \mathscr {H}_{G,k}^\mathrm{col}\right) , \end{aligned}$$

since $$1-(x_i^c)^2 = \left( 1-x_i^c\right) ^2 + 2 \left( x_i^c - (x_i^c)^2\right) $$, and

28 $$\begin{aligned} x_i^c-\left( x_i^c\right) ^2= x^c_i\left( 1-\sum _{c'}x^{c'}_i\right) +\sum _{c' : c'\ne c}x^c_ix^{c'}_i \in \mathscr {I}_2\left( \mathscr {H}^\mathrm{col}_{G,k}\right) , \end{aligned}$$

and the analogous statements hold for $\mathscr {H}^\mathrm {stab}_{G,k}$. Hence, both ${\mathscr {M}}(\emptyset ) + {\mathscr {I}}\left( \mathscr {H}_k^\mathrm{col}\right)$ and ${\mathscr {M}}(\emptyset ) + {\mathscr {I}}\left( \mathscr {H}_k^\mathrm{stab}\right)$ are Archimedean and we can apply Theorems 2 and 3 to express the quantum graph parameters in terms of positive tracial linear functionals. Namely,

$$\begin{aligned} \chi _{qc}(G)&= \min \left\{ k \in \mathbb {N}: L \in \mathbb {R}\langle \{x_i^c: i \in V, c \in [k]\} \rangle ^* \text { symmetric, tracial, positive,}\right. \\&\qquad \quad \,\,\quad \qquad \qquad \left. L(1) = 1, \, L=0 \text { on } {\mathscr {I}}(\mathscr {H}_{G,k}^\mathrm{col})\right\} , \end{aligned}$$

and $\chi _q(G)$ is obtained by adding the constraint ${{\mathrm{rank}}}(M(L)) < \infty$. Likewise,

$$\begin{aligned} \alpha _{qc}(G)&= \max \left\{ k \in \mathbb {N}: L \in \mathbb {R}\langle \{x_c^i: c \in [k], i \in V\} \rangle ^* \text { symmetric, tracial, positive,}\right. \\&\qquad \qquad \quad \quad \,\,\,\,\,\,\,\, \quad \left. L(1) = 1, \, L=0 \text { on } {\mathscr {I}}(\mathscr {H}_{G,k}^\mathrm{stab}) \right\} , \end{aligned}$$

and $\alpha _q(G)$ is given by this program with the additional constraint ${{\mathrm{rank}}}(M(L)) <\infty$.

Starting from these formulations it is natural to define a hierarchy $\smash \{\gamma _r^\mathrm {col}(G)\}$ of lower bounds on $\chi _{qc}(G)$ and a hierarchy $\smash \{\gamma _r^\mathrm {stab}(G)\}$ of upper bounds on $\alpha _{qc}(G)$, where the bounds of order $r\in \mathbb {N}$ are obtained by truncating *L* to polynomials of degree at most 2*r* and truncating the ideal to degree 2*r*:

$$\begin{aligned} \gamma ^\mathrm {col}_r(G)&= \min \left\{ k \in \mathbb {N}: L \in \mathbb {R}\langle \{x_i^c: i \in V, c \in [k]\} \rangle _{2r}^* \text { symmetric, tracial, positive,} \right. \\&\qquad \qquad \quad \quad \qquad \left. L(1) = 1, \, L=0 \text { on } {\mathscr {I}}_{2r}\left( \mathscr {H}_{G,k}^\mathrm{col}\right) \right\} , \\ \gamma ^{\mathrm {stab}}_{r}(G)&= \max \left\{ k \in \mathbb {N}: L \in \mathbb {R}\langle \{x_c^i: c \in [k], i \in V\} \rangle _{2r}^* \text { symmetric, tracial, positive,} \right. \\&\qquad \qquad \quad \quad \, \qquad \left. L(1) = 1, \, L=0 \text { on } {\mathscr {I}}_{2r}(\mathscr {H}_{G,k}^\mathrm{stab}) \right\} . \end{aligned}$$

Then, by defining $\gamma ^\mathrm{col}_*(G)$ and $\gamma ^\mathrm{stab}_*(G)$ by adding the constraint $\mathrm {rank}(M(L)) < \infty$ to $\gamma ^\mathrm{col}_\infty (G)$ and $\gamma ^\mathrm{stab}_\infty (G)$, we have

$$\begin{aligned} \gamma ^\mathrm{col}_\infty (G)= & {} \chi _{qc}(G), \ \ \gamma ^\mathrm{stab}_\infty (G)=\alpha _{qc}(G), \ \text {and} \\ \gamma ^\mathrm{col}_*(G)= & {} \chi _q(G), \ \ \quad \gamma ^\mathrm{stab}_*(G) = \alpha _q(G). \end{aligned}$$

The optimization problems $\gamma _r^\mathrm{col}(G)$, for $r \in \mathbb {N}$, can be computed by semidefinite programming and binary search on *k*, since the positivity condition on *L* can be expressed by requiring that its truncated moment matrix $M_r(L)=(L(w^*w'))$ (indexed by words with degree at most *r*) is positive semidefinite. If there is an optimal solution (*k*, *L*) to $\gamma ^\mathrm{col}_r(G)$ with *L* flat, then, by Theorem 4, we have equality $\gamma _r^\mathrm {col}(G) = \chi _q(G)$. Since $\smash \{\gamma _r^\mathrm {col}(G)\}_{r\in \mathbb {N}}$ is a monotone nondecreasing sequence of lower bounds on $\chi _q(G)$, there exists an $r_0$ such that for all $r \ge r_0$ we have $\gamma _r^\mathrm {col}(G) = \gamma _{r_0}^\mathrm {col}(G)$, which is equal to $\gamma ^\mathrm{col}_\infty (G) = \chi _{qc}(G)$ by Lemma 1. The analogous statements hold for the parameters $\gamma _r^\mathrm {stab}(G)$. Hence, we have shown the following result.

#### Proposition 8

There is an $r_0 \in \mathbb {N}$ such that $\gamma _r^\mathrm {col}(G) = \chi _{qc}(G)$ and $\gamma _r^\mathrm {stab}(G) = \alpha _{qc}(G)$ for all $r \ge r_0$. Moreover, if $\gamma _r^\mathrm {col}(G)$ admits a flat optimal solution, then $\gamma _r^\mathrm {col}(G) = \chi _q(G)$, and if $\gamma _r^\mathrm {stab}(G)$ admits a flat optimal solution, then $\gamma _r^\mathrm {stab}(G) = \alpha _q(G)$.

#### Remark 2

A hierarchy $\{\mathscr {Q}_r(\varGamma )\}$ of semidefinite outer approximations for the set $C_{qc}(\varGamma )$ of commuting quantum correlations was constructed in [46] (revisiting the approach in [36, 48]). This hierarchy converges, that is,

$$\begin{aligned} C_{qc}(\varGamma )=\mathscr {Q}_{\infty }(\varGamma )=\bigcap _{r\in \mathbb {N}}\mathscr {Q}_{r}(\varGamma ). \end{aligned}$$

These approximations $\mathscr {Q}_r(\varGamma )$ are based on the eigenvalue optimization approach, applied to the formulation (4) of commuting quantum correlations. So they use linear functionals on polynomials involving the two sets of variables $x_s^a$ and $y_t^b$ for $(a,b,s,t)\in \varGamma$. Paulsen et al. [46] use these outer approximations to define a hierarchy of lower bounds converging to $\chi _{qc}(G)$, where the bounds are defined in terms of feasibility problems over the sets $\mathscr {Q}_r(\varGamma )$.

For synchronous correlations we can use the result of Theorem 5 and the tracial optimization approach used here to define directly a converging hierarchy $\{\mathscr {Q}_{r,s}(\varGamma )\}$ of outer semidefinite approximations for the set $C_{qc,s}(\varGamma )$ of synchronous commuting quantum correlations. These approximations now use linear functionals on polynomials involving only one set of variables $x_s^a$ for $(a,s)\in A \times S$. Namely, for $r\in \mathbb {N}\cup \{\infty \}$ define $\mathscr {Q}_{r,s}(\varGamma )$ as the set of $P \in \mathbb {R}^{\varGamma }$ for which there exists a symmetric, tracial, positive linear functional $L\in \mathbb {R}\langle \{x_s^a: (a,s)\in A\times S\}\rangle _{2r}^*$ such that $L(1)=1$ and $L=0$ on the ideal generated by the polynomials $x_s^a-(x_s^a)^2$ ($(a,s)\in A\times S$) and $1-\sum _{a\in A} x_s^a$ ($s\in S$), truncated at degree 2*r*. Then we have

$$\begin{aligned} C_{qc,s}(\varGamma )=\mathscr {Q}_{\infty ,s}(\varGamma )=\bigcap _{r\in \mathbb {N}}\mathscr {Q}_{r,s}(\varGamma ). \end{aligned}$$

The *synchronous value* of a nonlocal game is defined in [13] as the maximum value of the objective function (11) over the set $C_{qc,s}(\varGamma )$. By maximizing the objective (11) over the relaxations $\mathscr {Q}_{r,s}(\varGamma )$ we get a hierarchy of semidefinite programming upper bounds that converges to the synchronous value of the game. Finally note that one can also view the parameters $\gamma _r^\mathrm {col}(G)$ as solving feasibility problems over the sets $\mathscr {Q}_{r,s}(\varGamma )$.

### Hierarchies $\xi _r^\mathrm {col}(G)$ and $\xi _r^\mathrm {stab}(G)$ based on Lasserre type bounds

Here we revisit some known Lasserre type hierarchies for the classical stability number $\alpha (G)$ and chromatic number $\chi (G)$ and we show that their tracial noncommutative analogues can be used to recover known parameters such as the projective packing number $\alpha _p(G)$, the projective rank $\xi _f(G)$, and the tracial rank $\xi _\mathrm{tr}(G)$. Compared to the hierarchies defined in the previous section, these Lasserre type hierarchies use less variables (they only use variables indexed by the vertices of the graph *G*), but they also do not converge to the (commuting) quantum chromatic or stability number.

Given a graph $G=(V,E)$, define the set of polynomials

$$\begin{aligned} \mathscr {H}_G = \big \{ x_i - x_i^2: i \in V\big \} \cup \big \{ x_i x_j: \{i,j\} \in E\big \} \end{aligned}$$

in the variables $\mathbf x=(x_i: i\in V)$ (which are commutative or noncommutative depending on the context). Note that $1-x_i^2\in {\mathscr {M}}_2(\emptyset ) + \mathscr {I}_2(\mathscr {H}_G)$ for all $i\in V$, so that ${\mathscr {M}}(\emptyset ) + \mathscr {I}(\mathscr {H}_G)$ is Archimedean.

#### Semidefinite programming bounds on the projective packing number

We first recall the Lasserre hierarchy of bounds for the classical stability number $\alpha (G)$. Starting from the formulation of $\alpha (G)$ via the optimization problem

29 $$\begin{aligned} \alpha (G) = \sup \left\{ \sum _{i \in V} x_i : x\in \mathbb {R}^n, \ h(x) = 0 \text { for } h \in \mathscr {H}_G\right\} , \end{aligned}$$

the *r*-th level of the Lasserre hierarchy for $\alpha (G)$ (introduced in [24, 26]) is defined by

$$\begin{aligned} {\mathrm {las}_{r}^{\mathrm {stab}}}(G)= \mathrm {sup} \left\{ L\left( \sum _{i \in V} x_i\right) : L\in \mathbb {R}[\mathbf {x}]_{2r}^* \text { positive}, \, L(1)=1,\, L= 0 \text { on } {\mathscr {I}}_{2r}(\mathscr {H}_G)\right\} . \end{aligned}$$

Then we have ${\mathrm {las}_{r+1}^{\mathrm {stab}}}(G) \le {\mathrm {las}_{r}^{\mathrm {stab}}}(G)$ and the first bound is Lovász’ theta number: ${\mathrm {las}_{1}^{\mathrm {stab}}}(G)=\vartheta (G)$. Finite convergence to $\alpha (G)$ is shown in [26]:

$$\begin{aligned} {\mathrm {las}_{\alpha (G)}^{\mathrm {stab}}}(G) = \alpha (G). \end{aligned}$$

Roberson [51] introduces the *projective packing number*

30 $$\begin{aligned} \alpha _p(G)&= \sup \left\{ \frac{1}{d}\sum _{i \in V} {{\mathrm{rank}}}X_i : d \in \mathbb {N},\, \mathbf{X} \in (\mathscr {S}^d)^n \text { projectors}, \ \right. \nonumber \\&\qquad \qquad \qquad \qquad \qquad \quad \quad \,\left. X_i X_j = 0 \text { for } \{i,j\} \in E \right\} \nonumber \\&= \mathrm {sup}\Big \{\frac{1}{d}\mathrm {Tr}\Big (\sum _{i \in V} X_i \Big ) : d\in \mathbb {N},\, \mathbf{X} \in (\mathscr {S}^d)^n, \, h(\mathbf{X}) = 0 \text { for } h\in \mathscr {H}_G \Big \} \end{aligned}$$

as an upper bound for the quantum stability number $\alpha _q(G)$. Here $\mathscr {S}^d$ denotes the set of real symmetric $d\times d$ matrices. Note that the inequality $\alpha _q(G)\le \alpha _p(G)$ also follows from Proposition 9 below. Comparing (29) and (30) we see that the parameter $\alpha _p(G)$ can be viewed as a noncommutative analogue of $\alpha (G)$.

For $r \in \mathbb {N}\cup \{\infty \}$ we define the noncommutative analogue of ${\mathrm {las}_{r}^{\mathrm {stab}}}(G)$ by

$$\begin{aligned} {\xi _{r}^{\mathrm {stab}}}(G) = \mathrm {sup}\Big \{L\Big (\sum _{i \in V} x_i\Big ) : \;&L\in \mathbb {R}\langle \mathbf{x}\rangle _{2r}^* \text { tracial, symmetric, and positive}, \\&L(1)=1,\, L = 0 \text { on } {\mathscr {I}}_{2r}(\mathscr {H}_G) \Big \}, \end{aligned}$$

and ${\xi _{*}^{\mathrm {stab}}}(G)$ by adding the constraint ${{\mathrm{rank}}}(M(L)) < \infty$ to the definition of ${\xi _{\infty }^{\mathrm {stab}}}(G)$.

In view of Theorems 2 and 3, both ${\xi _{\infty }^{\mathrm {stab}}}(G)$ and ${\xi _{*}^{\mathrm {stab}}}(G)$ can be reformulated in terms of $C^*$-algebras: ${\xi _{\infty }^{\mathrm {stab}}}(G)$ (resp., ${\xi _{*}^{\mathrm {stab}}}(G)$) is the largest value of $\tau (\sum _{i\in V}X_i)$, where ${\mathscr {A}}$ is a (resp., finite-dimensional) $C^*$-algebra with tracial state $\tau$ and $X_i \in {\mathscr {A}}$ ($i \in [n]$) are projectors satisfying $X_i X_j = 0$ for all $\{i,j\} \in E$. Moreover, as we now see, the parameter ${\xi _{*}^{\mathrm {stab}}}(G)$ coincides with the projective packing number and the parameters ${\xi _{*}^{\mathrm {stab}}}(G)$ and ${\xi _{\infty }^{\mathrm {stab}}}(G)$ upper bound the quantum stability numbers.

##### Proposition 9

We have ${\xi _{*}^{\mathrm {stab}}}(G) = \alpha _p(G)\ge \alpha _q(G)$ and ${\xi _{\infty }^{\mathrm {stab}}}(G)\ge \alpha _{qc}(G)$.

##### Proof

By  (30), $\alpha _p(G)$ is the largest value of $L(\sum _{i\in V}x_i)$ taken over all linear functionals *L* that are normalized trace evaluations at projectors $\mathbf X\in (\S ^d)^n$ (for some $d \in \mathbb {N}$) with $X_i X_j = 0$ for $\{i,j\} \in E$. By convexity the optimum remains unchanged when considering a convex combination of such trace evaluations. In view of Theorem 3 [the equivalence between (1) and (3)], we can conclude that this optimum value is precisely the parameter ${\xi _{*}^{\mathrm {stab}}}(G)$. This shows equality $\alpha _p(G)={\xi _{*}^{\mathrm {stab}}}(G)$.

Consider a $C^*$-algebra $\mathscr {A}$ with tracial state $\tau$ and a set of projectors $X^i_c\in \mathscr {A}$ (for $i\in V,\ c\in [k]$) satisfying (26)–(27). Then, setting $X_i=\sum _{c\in [k]} X^i_c$ for $i\in V$, we obtain projectors $X_i\in \mathscr {A}$ that satisfy $X_iX_j=0$ if $\{i,j\}\in E$. Moreover, the following holds: $\tau (\sum _{i\in V}X_i)=\sum _{c\in [k]} \tau (\sum _{i\in V}X^i_c)=k$. This shows ${\xi _{\infty }^{\mathrm {stab}}}(G)\ge \alpha _{qc}(G)$ and, when restricting $\mathscr {A}$ to be finite dimensional, ${\xi _{*}^{\mathrm {stab}}}(G)\ge \alpha _q(G)$. $\square$

Using Lemma 1 one can verify that ${\xi _{r}^{\mathrm {stab}}}(G)$ converges to ${\xi _{\infty }^{\mathrm {stab}}}(G)$ as $r~\rightarrow ~\infty$, and for $r \in \mathbb {N}\cup \{\infty \}$ the infimum in ${\xi _{r}^{\mathrm {stab}}}(G)$ is attained. Moreover, by Theorem 4, if ${\xi _{r}^{\mathrm {stab}}}(G)$ admits a flat optimal solution, then equality ${\xi _{r}^{\mathrm {stab}}} = {\xi _{*}^{\mathrm {stab}}}(G)$ holds. The first bound ${\xi _{1}^{\mathrm {stab}}}(G)$ coincides with the theta number, since ${\xi _{1}^{\mathrm {stab}}}(G)={\mathrm {las}_{1}^{\mathrm {stab}}}(G)=\vartheta (G)$. Summarizing we have $\alpha _{qc}(G)\le {\xi _{\infty }^{\mathrm {stab}}}(G)$ and the following chain of inequalities

$$\begin{aligned} \alpha _q(G)\le \alpha _p(G)={\xi _{*}^{\mathrm {stab}}}(G)\le {\xi _{\infty }^{\mathrm {stab}}}(G)\le {\xi _{r}^{\mathrm {stab}}}(G)\le {\xi _{1}^{\mathrm {stab}}}(G)=\vartheta (G). \end{aligned}$$

#### Semidefinite programming bounds on the projective rank and tracial rank

We now turn to the (quantum) chromatic numbers. First recall the definition of the fractional chromatic number:

$$\begin{aligned} \chi _f(G) := \min \Big \{ \sum _{S \in \mathscr {S}_{G}} \lambda _S : \lambda \in \mathbb {R}_+^{ \mathscr {S}_{G}},\, \sum _{S\in \mathscr {S}_{G}: i\in S} \lambda _S = 1 \text { for all } i\in V\Big \}, \end{aligned}$$

where $\mathscr {S}_{G}$ is the set of stable sets of *G*. Clearly, $\chi _f(G)\le \chi (G)$. The following Lasserre type lower bounds for the classical chromatic number $\chi (G)$ are defined in [19]:

$$\begin{aligned} {\mathrm {las}_{r}^{\mathrm {col}}}(G)= & {} \mathrm {inf} \left\{ L(1) : L\in \mathbb {R}[\mathbf {x}]_{2r}^* \text { positive},\, L(x_i)=1\ (i \in V),\right. \\&\quad \qquad \qquad \quad L = \left. 0 \text { on } \mathscr {I}_{2r}(\mathscr {H}_G)\right\} . \end{aligned}$$

Note that we may view $\chi _f(G)$ as minimizing *L*(1) over all linear functionals $L\in \mathbb {R}[\mathbf {x}]^*$ that are conic combinations of evaluations at characteristic vectors of stable sets. From this we see that ${\mathrm {las}_{r}^{\mathrm {col}}}(G)\le \chi _f(G)$ for all $r \ge 1$. In [19] it is shown that finite convergence to $\chi _f(G)$ holds:

$$\begin{aligned} {\mathrm {las}_{\alpha (G)}^{\mathrm {col}}}(G) = \chi _f(G). \end{aligned}$$

The bound of order $r=1$ coincides with the theta number: ${\mathrm {las}_{1}^{\mathrm {col}}}(G)=\vartheta (\overline{G})$.

The following parameter $\xi _f(G)$, called the *projective rank* of *G*, was introduced in [32] as a lower bound on the quantum chromatic number $\chi _q(G)$:

$$\begin{aligned} \xi _f(G) := \mathrm {inf}&\left\{ \frac{d}{r} : d,r\in \mathbb {N},\ X_1, \ldots , X_n \in \mathscr {S}^d, \ {{\mathrm{Tr}}}(X_i)=r\ (i\in V), \right. \\&\left. \qquad \ X_i^2 = X_i \ (i \in V), \ X_i X_j = 0\ (\{i,j\} \in E) \right\} . \end{aligned}$$

##### Proposition 10

([32]) For any graph *G* we have $\xi _f(G)\le \chi _q(G)$.

##### Proof

Set $k=\chi _q(G)$. It is shown in [9] that in the definition of $\chi _q(G)$ from (24)–(25), one may assume w.l.o.g. that $X^c_i$ are projectors that all have the same rank, say, *r*. Then, for any given color $c\in [k]$, the matrices $X^c_i$ ($i\in V$) provide a feasible solution to $\xi _f(G)$ with value *d* / *r*. This shows $\xi _f(G)\le d/r$. Finally, $d/r=k$ holds since by (24)–(25) we have $d={{\mathrm{rank}}}(I)=\sum _{c=1}^k{{\mathrm{rank}}}(X^c_i)= kr$. $\square$

In [46, Prop. 5.11] it is shown that the projective rank can equivalently be defined as

$$\begin{aligned} \xi _f(G)&= \mathrm {inf} \big \{ \lambda : \; \mathscr {A} \text { is a finite dimensional } C^*\text {-algebra with tracial state } \tau ,\\&\qquad \qquad \qquad X_i \in \mathscr {A} \text { projector with } \tau (X_i) = 1/\lambda \, (i\in V),\\&\qquad \qquad \qquad X_i X_j = 0 \ (\{i,j\} \in E)\big \}. \end{aligned}$$

Paulsen et al. [46] also define the *tracial rank* $\xi _{tr}(G)$ of *G* as the parameter obtained by omitting in the above definition of $\xi _f(G)$ the restriction that $\mathscr {A}$ has to be finite dimensional. The motivation for the parameter $\xi _{tr}(G)$ is that it lower bounds the *commuting* quantum chromatic number [46, Thm. 5.11]:

$$\begin{aligned} \xi _{tr}(G)\le \chi _{qc}(G). \end{aligned}$$

Using Theorems 2 and 3 (which we apply to *L* / *L*(1) when *L* is not normalized), we obtain the following reformulations:

$$\begin{aligned} \xi _{f}(G)&= \mathrm {inf} \left\{ L(1) : L\in \mathbb {R}\langle \mathbf{x}\rangle ^* \text { tracial, symmetric, positive}, \, {{\mathrm{rank}}}(M(L))<\infty ,\right. \\&\qquad \qquad \,\quad \qquad \left. L(x_i)=1\ (i \in V),\, L = 0 \text { on } \mathscr {I}(\mathscr {H}_G) \right\} , \end{aligned}$$

and $\xi _{tr}(G)$ is obtained by the same program without the restriction ${{\mathrm{rank}}}(M(L)) < \infty$. In addition, we obtain that in this formulation of $\xi _f(G)$ we can equivalently optimize over all *L* that are conic combinations of trace evaluations at projectors $X_i \in \S ^d$ (for some $d \in \mathbb {N}$) satisfying $X_i X_j = 0$ for all $\{i,j\} \in E$. If we restrict the optimization to conic combinations of *scalar* evaluations ($d=1$) we obtain the fractional chromatic number. This shows that the projective rank can be seen as the noncommutative analogue of the fractional chromatic number, as was already observed in [32, 46].

The above formulations of the parameters $\xi _{tr}(G)$ and $\xi _f(G)$ in terms of linear functionals also show that they fit within the following hierarchy $\smash {\{{\xi _{r}^{\mathrm {col}}}(G)\}_{r\in \mathbb {N}\cup \{\infty \}}}$, defined as the noncommutative tracial analogue of the hierarchy $\{{\mathrm {las}_{r}^{\mathrm {col}}}(G)\}_{r}$:

$$\begin{aligned} {\xi _{r}^{\mathrm {col}}}(G)&= \mathrm {inf} \left\{ L(1) : L\in \mathbb {R}\langle \mathbf{x}\rangle _{2r}^* \text { tracial, symmetric, and positive}, \right. \\&\left. \quad \qquad \qquad \qquad L(x_i)=1\ (i \in V),\, L = 0 \text { on } {\mathscr {I}}_{2r}(\mathscr {H}_G) \right\} . \end{aligned}$$

Again, ${\xi _{*}^{\mathrm {col}}}(G)$ is the parameter obtained by adding the constraint ${{\mathrm{rank}}}(M(L)) <\infty$ to the program defining ${\xi _{\infty }^{\mathrm {col}}}(G)$. By the above discussion the following holds.

##### Proposition 11

We have ${\xi _{*}^{\mathrm {col}}}(G) = \xi _f(G)\le \chi _q(G)$ and ${\xi _{\infty }^{\mathrm {col}}}(G)=\xi _{tr}(G)\le \chi _{qc}(G)$.

Using Lemma 1 one can verify that the parameters ${\xi _{r}^{\mathrm {col}}}(G)$ converge to ${\xi _{\infty }^{\mathrm {col}}}(G)$. Moreover, by Theorem 4, if ${\xi _{r}^{\mathrm {col}}}(G)$ admits a flat optimal solution, then we have ${\xi _{r}^{\mathrm {col}}} = {\xi _{*}^{\mathrm {col}}}(G)$. Also, the parameter ${\xi _{1}^{\mathrm {col}}}(G)$ coincides with ${\mathrm {las}_{1}^{\mathrm {col}}}(G)=\vartheta (\overline{G})$. Summarizing we have ${\xi _{\infty }^{\mathrm {col}}}(G)=\xi _{tr}(G)\le \chi _{qc}(G)$ and the following chain of inequalities

$$\begin{aligned} \vartheta (\overline{G})={\xi _{1}^{\mathrm {col}}}(G) \le {\xi _{r}^{\mathrm {col}}}(G)\le {\xi _{\infty }^{\mathrm {col}}}(G)=\xi _{tr}(G)\le {\xi _{*}^{\mathrm {col}}}(G)=\xi _f(G) \le \chi _q(G). \end{aligned}$$

Observe that the bounds ${\mathrm {las}_{r}^{\mathrm {col}}}(G)$ and ${\xi _{r}^{\mathrm {col}}}(G)$ remain below the fractional chromatic number $\chi _f(G)$, since $\xi _f(G)= {\xi _{*}^{\mathrm {col}}}(G)\le {\mathrm {las}_{*}^{\mathrm {col}}}(G)=\chi _f(G)$. Hence, these bounds are weak if $\chi _f(G)$ is close to $\vartheta (\overline{G})$ and far from $\chi (G)$ or $\chi _q(G)$. In the classical setting this is the case, e.g., for the class of Kneser graphs $G=K(n,r)$, with vertex set the set of all *r*-subsets of [*n*] and having an edge between any two disjoint *r*-subsets. By results of Lovász [29, 30], the fractional chromatic number is *n* / *r*, which is known to be equal to $\vartheta (\overline{K(n,r)})$, while the chromatic number is $n-2r+2$. In [19] this was used as a motivation to define a new hierarchy of lower bounds $\{\varLambda _r(G)\}$ on the chromatic number that can go beyond the fractional chromatic number. In Sect. 4.3 we recall this approach and show that its extension to the tracial setting recovers the hierarchy $\{\gamma _r^\mathrm {col}(G)\}$ introduced in Sect. 4.1. We also show how a similar technique can be used to recover the hierarchy $\{\gamma _r^\mathrm {stab}(G)\}$.

#### A link between ${\xi _{r}^{\mathrm {stab}}}(G)$ and ${\xi _{r}^{\mathrm {col}}}(G)$

In [19, Thm. 3.1] it is shown that the bounds ${\mathrm {las}_{r}^{\mathrm {stab}}}(G)$ and ${\mathrm {las}_{r}^{\mathrm {col}}}(G)$ satisfy

$$\begin{aligned} {\mathrm {las}_{r}^{\mathrm {stab}}}(G) {\mathrm {las}_{r}^{\mathrm {col}}}(G) \ge |V|\quad \text { for any } r\ge 1, \end{aligned}$$

with equality if *G* is vertex-transitive. This extends a well-known property of the theta number (i.e., the case $r=1$). The same property holds for the noncommutative analogues ${\xi _{r}^{\mathrm {stab}}}(G)$ and ${\xi _{r}^{\mathrm {col}}}(G)$.

##### Lemma 2

For a graph $G=(V,E)$ and $r\in \mathbb {N}\cup \{\infty ,*\}$ we have ${\xi _{r}^{\mathrm {stab}}}(G){\xi _{r}^{\mathrm {col}}}(G)\ge |V|,$ with equality if *G* is vertex-transitive.

##### Proof

Let *L* be feasible for ${\xi _{r}^{\mathrm {col}}}(G)$. Then $\tilde{L} = L/L(1)$ provides a solution to ${\xi _{r}^{\mathrm {stab}}}(G)$ with value $\tilde{L}\big (\sum _{i\in V}x_i\big )= |V|/L(1)$, implying that ${\xi _{r}^{\mathrm {stab}}}(G)\ge |V|/L(1)$ and therefore ${\xi _{r}^{\mathrm {stab}}}(G){\xi _{r}^{\mathrm {col}}}(G)\ge |V|$.

Assume *G* is vertex-transitive. Let *L* be a feasible solution for ${\xi _{r}^{\mathrm {stab}}}(G)$. As *G* is vertex-transitive we may assume (after symmetrization) that $L(x_i)$ takes a constant value. Set $L(x_i)=:1/\lambda$ for all $i\in V$, so that the objective value of *L* for ${\xi _{r}^{\mathrm {stab}}}(G)$ is $|V|/\lambda$. Then $\tilde{L} = \lambda L$ provides a feasible solution for ${\xi _{r}^{\mathrm {col}}}(G)$ with value $\lambda$, implying ${\xi _{r}^{\mathrm {col}}}(G) \le \lambda$. This shows ${\xi _{r}^{\mathrm {col}}}(G){\xi _{r}^{\mathrm {stab}}}(G)\le |V|$. $\square$

For a vertex-transitive graph *G*, the inequality $\xi _f(G)\alpha _q(G) \le |V|$ is shown in [32, Lem. 6.5]; it can be recovered from the $r=*$ case of Lemma 2 and $\alpha _q(G) \le \alpha _p(G)$.

#### Comparison to existing semidefinite programming bounds

By adding the constraints $L(x_i x_j) \ge 0$, for all $i,j \in V$, to the program defining ${\xi _{1}^{\mathrm {col}}}(G)$, we obtain the strengthened theta number $\vartheta ^+(\overline{G})$ (from [56]). Moreover, if we add the constraints

31 $$\begin{aligned} L(x_ix_j)&\ge 0 \quad \text { for }\quad i\ne j\in V, \end{aligned}$$

32 $$\begin{aligned} \sum _{j\in C} L(x_ix_j)&\le 1 \quad \text { for }\quad i\in V, \end{aligned}$$

33 $$\begin{aligned} \mathbf L(1) + \sum _{i\in C, j\in C'}L(x_ix_j)&\ge |C| + |C'| \quad \text { for } \quad C,C' \text { distinct cliques in } G \end{aligned}$$

to the program defining the parameter ${\xi _{1}^{\mathrm {col}}}(G)$, then we obtain the parameter $\xi _\mathrm {SDP}(G)$, which is introduced in [46, Thm. 7.3] as a lower bound on $\xi _\mathrm {tr}(G)$. We will now show that the inequalities (31)–(33) are in fact valid for ${\xi _{2}^{\mathrm {col}}}(G)$, which implies

$$\begin{aligned} {\xi _{2}^{\mathrm {col}}}(G) \ge \xi _\mathrm {SDP}(G) \ge \vartheta ^+(\overline{G}). \end{aligned}$$

For this, given a clique *C* in *G*, we define the polynomial

$$\begin{aligned} g_C:=1-\sum _{i\in C}x_i\in \mathbb {R}\langle \mathbf{x}\rangle . \end{aligned}$$

Then (32) and (33) can be reformulated as $L(x_i g_C) \ge 0$ and $L(g_C g_{C'}) \ge 0$, respectively, using the fact that $L(x_i) = L(x_i^2)=1$ for all $i \in V$. Hence, to show that any feasible *L* for ${\xi _{2}^{\mathrm {col}}}(G)$ satisfies  (31)–(33), it suffices to show Lemma 3 below. Recall that a commutator is a polynomial of the form $[p,q]=pq-qp$ with $p,q\in \mathbb {R}\langle \mathbf{x}\rangle$. We denote by $\varTheta _{r}$ the set of linear combinations of commutators [*p*, *q*] with $\deg (pq)\le r$.

##### Lemma 3

Let *C* and $C'$ be cliques in a graph *G* and let $i,j\in V$. Then we have

$$\begin{aligned} g_C \in {\mathscr {M}}_2(\emptyset )+{\mathscr {I}}_2(\mathscr {H}_G), \text { and } \ x_ix_j,\ x_ig_C,\ g_Cg_{C'} \in {\mathscr {M}}_4(\emptyset )+\mathscr {I}_4(\mathscr {H}_G)+\varTheta _4. \end{aligned}$$

##### Proof

The claim $g_C\in {\mathscr {M}}_2(\emptyset )+{\mathscr {I}}_2(\mathscr {H}_G)$ follows from the identity

34 $$\begin{aligned} g_C=\Big (\underbrace{ 1-\sum _{i\in C}x_i}_{g_C}\Big )^2 +\underbrace{\sum _{i\in C} (x_i-x_i^2) +\sum _{i\ne j\in C} x_ix_j}_{h}=g_C^2+h, \end{aligned}$$

where $h\in \mathscr {I}_2(\mathscr {H}_G)$. We also have

$$\begin{aligned} x_ix_j&= x_ix_j^2x_i +x_j(x_i-x_i^2)+x_i^2(x_j-x_j^2) +[x_i,x_ix_j^2]+[x_i-x_i^2,x_j], \\ x_ig_C&= x_ig_C^2x_i+ g_C^2(x_i-x_i^2) + [x_i-x_i^2,g_C^2] +[x_i,x_ig_C^2], \end{aligned}$$

and, writing analogously $g_{C'} =g_{C'}^2+h'$ with $h'\in \mathscr {I}_2(\mathscr {H}_G)$, we have

$$\begin{aligned} g_Cg_{C'}= g_C g_{C'}^2 g_C + [g_C,g_C g_{C'}^2]+[h,g_{C'}^2]+g_C^2 h'+hh'+ g_{C'}^2 h. \end{aligned}$$

$$\square $$

Using the bound $\xi _\mathrm{{SDP}}(G)$ it is shown in [46, Thm. 7.4] that the tracial rank of the cycle $C_{2n+1}$ satisfies ${\xi _{\infty }^{\mathrm {col}}}(C_{2n+1})=(2n+1)/n$. Combining this with Lemma 2 gives $n = {\xi _{\infty }^{\mathrm {stab}}}(C_{2n+1}) \ge \alpha _{qc}(C_{2n+1})$, and equality holds since $\alpha _{qc}(C_{2n+1})\ge \alpha (C_{2n+1})=n$.

### Links between the bounds $\gamma ^\mathrm{col}_r(G)$, $\xi _r^\mathrm {col}(G)$, $\gamma ^\mathrm{stab}_r(G)$, and $\xi _r^\mathrm {stab}(G)$

In this last section, we make the link between the two hierarchies $\{{\xi _{r}^{\mathrm {stab}}}(G)\}$ (resp. $\{{\xi _{r}^{\mathrm {col}}}(G)\}$) and $\{\gamma ^\mathrm{stab}_r(G)\}$ (resp. $\{\gamma ^\mathrm{col}_r(G)\}$). The key tool is the interpretation of the coloring and stability numbers in terms of certain graph products.

We start with the (quantum) coloring number. For an integer *k*, recall that the Cartesian product $G\Box K_k$ of *G* and the complete graph $K_k$ is the graph with vertex set $V\times [k]$, where two vertices (*i*, *c*) and $(j,c')$ are adjacent if ($\{i,j\}\in E$ and $c=c'$) or ($i=j$ and $c\ne c'$). The following is a well-known reduction of the chromatic number $\chi (G)$ to the stability number of the Cartesian product $G\Box K_k$:

$$\begin{aligned} \chi (G)=\min \big \{k\in \mathbb {N}: \alpha (G \square K_k)=|V|\big \}. \end{aligned}$$

It was used in [19] to define the following lower bounds on the chromatic number:

$$\begin{aligned} \varLambda _r(G) = \min \big \{ k\in \mathbb {N}: {\mathrm {las}_{r}^{\mathrm {stab}}}( G\Box K_k) = |V|\big \}, \end{aligned}$$

where it was also shown that ${\mathrm {las}_{r}^{\mathrm {col}}}(G) \le \varLambda _r(G)\le \chi (G)$ for all $r\ge 1$, with equality $\varLambda _{|V|}(G)=\chi (G)$. Hence the bounds $\varLambda _r(G)$ may go beyond the fractional chromatic number. This is the case for the above mentioned Kneser graphs; see [18] for other graph instances.

The above reduction from coloring to stability number has been extended to the quantum setting in [32], where it is shown that

$$\begin{aligned} \chi _q(G)=\min \{k\in \mathbb {N}: \alpha _q(G\Box K_k)=|V|\}. \end{aligned}$$

It is therefore natural to use the upper bounds ${\xi _{r}^{\mathrm {stab}}}(G\Box K_k)$ on $\alpha _q(G\Box K_k)$ in order to get the following lower bounds on the quantum coloring number:

35 $$\begin{aligned} \min \{k: {\xi _{r}^{\mathrm {stab}}}(G\Box K_k)=|V|\}, \end{aligned}$$

which are thus the noncommutative analogues of the bounds $\varLambda _r(G)$.

Observe that, for any $k\in \mathbb {N}$ and $r \in \mathbb {N}\cup \{\infty , *\}$, we have ${\xi _{r}^{\mathrm {stab}}}(G\Box K_k)\le |V|,$ which follows from Lemma 3 and the fact that the cliques $C_i=\{(i,c): c\in [k]\}$, for $i\in V$, cover all vertices in $G\Box K_k$. Let

$$\begin{aligned} \mathscr {C}_{G\Box K_k} = \big \{g_{C_i} : i\in V\big \}, \quad \text { where } \ g_{C_i} = 1-\sum _{c\in [k]}x^c_i, \end{aligned}$$

denote the set of polynomials corresponding to these cliques. We now show that the parameter (35) in fact coincides with the parameter $\gamma _r^\mathrm {col}(G)$ for all $r \in \mathbb {N}\cup \{\infty \}$.

For this observe first that the quadratic polynomials in the set $\smash {\mathscr {H}^\mathrm{col}_{G,k}}$ correspond precisely to the edges of $G\Box K_k$, and that the projector constraints are included in ${\mathscr {I}}_{2}(\mathscr {H}_{G,k}^\mathrm {col})$ [see (28)]. Hence we have

36 $$\begin{aligned} \smash {{\mathscr {I}}_{2r}(\mathscr {H}^\mathrm{col}_{G,k}) = \mathscr {I}_{2r}\left( \mathscr {H}_{G\Box K_k} \cup \mathscr {C}_{G\Box K_k}\right) }. \end{aligned}$$

We will also use the following result.

#### Lemma 4

Let $r\in \mathbb {N}\cup \{\infty ,*\}$ and assume *L* is feasible for ${\xi _{r}^{\mathrm {stab}}}(G\Box K_k)$. Then, we have $L(\sum _{i\in V, c\in [k]}x_i^c)=|V|$ if and only if $L=0$ on ${\mathscr {I}}_{2r}(\mathscr {C}_{G\Box K_k})$.

#### Proof

Assume $L=0$ on ${\mathscr {I}}_{2r}(\mathscr {C}_{G\Box K_k})$. Then $0=\sum _{i\in V}L(g_{C_i})= |V|- L(\sum _{i,c}x_i^c)$.

Conversely assume that $0= L\big (\sum _{i\in V, c\in [k]}x_i^c\big ) - |V|=\sum _{i\in V} L(g_{C_i})$. We will show $L=0$ on ${\mathscr {I}}_{2r}(\mathscr {C}_{G\Box K_k})$. For this we first observe that $g_{C_i}-(g_{C_i})^2\in {\mathscr {I}}_2(\mathscr {H}_{G\Box K_k})$ by (34). Hence $L(g_{C_i})=L(g_{C_i}^2)\ge 0$, which, combined with $\sum _i L(g_{C_i})=0$, implies $L(g_{C_i})=0$ for all $i\in V$. Next we show $L(wg_{C_i})=0$ for all words *w* with degree at most $2r-1$, using induction on $\deg (w)$. The base case $w=1$ holds by the above. Assume now $w=uv$, where $\deg (v)<\deg (u)\le r$. Using the positivity of *L*, the Cauchy-Schwarz inequality gives $|L(uvg_{C_i})| \le {L(u^*u)}^{1/2}{L(v^*g_{C_i}^2v)}^{1/2}$. Note that it suffices to show $L(v^*g_{C_i}v)=0$ since, using again (34), this implies $L(v^*g_{C_i}^2v)=0$ and thus $L(uvg_{C_i}) =0$. Using the tracial property of *L* and the induction assumption, we see that $L(v^*g_{C_i}v)=L(vv^*g_{C_i})=0$ since $\deg (vv^*)<\deg (w)$. $\square$

#### Proposition 12

For $r\in \mathbb {N}\cup \{\infty \}$ we have $\gamma ^\mathrm{col}_r(G)= \min \{k: {\xi _{r}^{\mathrm {stab}}}(G\Box K_k)=|V|\}.$

#### Proof

Let *L* be a linear functional certifying $\gamma ^\mathrm{col}_r(G) \le k$. Then, using (36) we see that *L* is feasible for $\smash {{\xi _{r}^{\mathrm {stab}}}(G\Box K_k)}$ and Lemma 4 shows that $L(\sum _{i,c}x_i^c)=|V|$. This shows ${\xi _{r}^{\mathrm {stab}}}(G\Box K_k)\ge |V|$ and thus equality holds (since the reverse inequality always holds). Therefore, $\min \{k: {\xi _{r}^{\mathrm {stab}}}(G\Box K_k) =|V|\}\le k$.

Conversely, assume ${\xi _{r}^{\mathrm {stab}}}(G\Box K_k)=|V|$. Since the optimum is attained, there exists a linear functional *L* feasible for ${\xi _{r}^{\mathrm {stab}}}(G\Box K_k)$ with $L(\sum _{i,c} x_i^c)=|V|$. Using Lemma 4 we can conclude that *L* is zero on $\smash {\mathscr {I}_{2r}(\mathscr {C}_{G\Box K_k})}$. Hence, in view of (36), *L* is zero on $\smash {\mathscr {I}_{2r}(\mathscr {H}^\mathrm{col}_{G,k})}$. This shows $\smash {\gamma ^\mathrm{col}_r(G)}\le k$. $\square$

Note that the proof of Proposition 12 also works in the commutative setting; this shows that the sequence $\varLambda _r(G)$ corresponds to the usual Lasserre hierarchy for the feasibility problem defined by the equations (24)–(25), which is another way of showing $\varLambda _\infty (G) = \chi (G)$.

We now turn to the (quantum) stability number. For $k \in \mathbb {N}$, consider the graph product $K_k\star G$, with vertex set $[k]\times G$, and with an edge between two vertices (*c*, *i*) and $(c',j)$ when $(c\ne c',i=j)$ or $(c=c', i\ne j)$ or $(c\ne c', \{i,j\}\in E)$. The product $K_k\star G$ coincides with the homomorphic product $K_k\ltimes \overline{G}$ used in [32, Sec. 4.2], where it is shown that

$$\begin{aligned} \alpha _q(G)=\max \big \{k \in \mathbb {N}: \alpha _q(K_k\star G)= k\big \}. \end{aligned}$$

This suggests using the upper bounds ${\xi _{r}^{\mathrm {stab}}}(K_k\star G)$ on $\alpha _q(K_k\star G)$ to define the following upper bounds on $\alpha _q(G)$:

37 $$\begin{aligned} \max \big \{k\in \mathbb {N}: {\xi _{r}^{\mathrm {stab}}}(K_k\star G)=k\big \}. \end{aligned}$$

For each $c \in [k]$, the set $C^c=\{(c,i):i\in V\}$ is a clique in $K_k\star G$, and we let

$$\begin{aligned} \mathscr {C}_{K_k\star G}=\big \{g_{C^c} : c\in [k]\big \},\quad \text { where }\ g_{C^c} = 1-\sum _{i\in V}x^i_c,\end{aligned}$$

denote the set of polynomials corresponding to these cliques. As these *k* cliques cover the vertex set of $K_k\star G$, we can use Lemma 3 to conclude that ${\xi _{r}^{\mathrm {stab}}}(K_k\star G)\le k$ for all $r\in \mathbb {N}\cup \{\infty ,*\}$.

Again, observe that the quadratic polynomials in the set $\mathscr {H}^\mathrm{stab}_{G,k}$ correspond precisely to the edges of $K_k\star G$ and that we have

$$\begin{aligned} \mathscr {I}_{2r}(\mathscr {H}^\mathrm{stab}_{G,k})= \mathscr {I}_{2r}(\mathscr {H}_{K_k\star G}\cup \mathscr {C}_{K_k\star G}). \end{aligned}$$

Based on this, one can show the analogue of Lemma 4: If *L* is feasible for the program ${\xi _{r}^{\mathrm {stab}}}(K_k\star G)$, then we have $L(\sum _{i,c}x^i_c)=k$ if and only if $L=0$ on $\mathscr {I}_{2r}(\mathscr {C}_{K_k\star G})$. This lemma can be used to show the following result, whose proof is analogous to that of Proposition 12 and thus omitted.

#### Proposition 13

For $r \in \mathbb {N}\cup \{\infty \}$ we have $\gamma ^\mathrm{stab}_r(G)= \max \{k: {\xi _{r}^{\mathrm {stab}}}(K_k\star G)=k\}.$

We do not know whether the results of Propositions 12 and 13 hold for $r=*$, because we do not know whether the supremum is attained in the program defining the parameter ${\xi _{*}^{\mathrm {stab}}}(\cdot )=\alpha _p(\cdot )$ (as was already observed in [51, p. 120]). Hence we can only claim the inequalities

$$\begin{aligned} \gamma ^\mathrm{col}_*(G)\ge & {} \min \{k: {\xi _{*}^{\mathrm {stab}}}(G\Box K_k)=|V|\} \quad \text {and} \quad \\ \gamma ^\mathrm{stab}_*(G)\le & {} \max \{k: {\xi _{*}^{\mathrm {stab}}}(K_k\star G)=k\}. \end{aligned}$$

As mentioned above, we have ${\mathrm {las}_{r}^{\mathrm {col}}}(G)\le \varLambda _r(G)$ for any $r\in \mathbb {N}$ [19, Prop. 3.3]. This result extends to the noncommutative setting and the analogous result holds for the stability parameters. In other words the hierarchies $\{\gamma ^\mathrm{col}_r(G)\}$ and $\{\gamma ^\mathrm{stab}_r(G)\}$ refine the hierarchies $\{{\xi _{r}^{\mathrm {col}}}(G)\}$ and $\{{\xi _{r}^{\mathrm {stab}}}(G)\}$.

#### Proposition 14

For $$r\in \mathbb {N}\cup \{\infty , *\}$$, $${\xi _{r}^{\mathrm {col}}}(G) \le \gamma ^\mathrm{col}_r(G)$$ and $$ {\xi _{r}^{\mathrm {stab}}}(G)\ge \gamma ^\mathrm{stab}_r(G)$$.

#### Proof

We may restrict to $r\in \mathbb {N}$ since we have seen earlier that the inequalities hold for $r\in \{\infty ,*\}$. The proof for the coloring parameters is similar to the proof of [19, Prop. 3.3] in the classical case and thus we omit it. We now show ${\xi _{r}^{\mathrm {stab}}}(G)\ge \gamma ^\mathrm{stab}_r(G)$. Set $k=\gamma ^\mathrm{stab}_r(G)$ and, using Proposition 13, let $L\in \mathbb {R}\langle x^i_c{:}\,i\in V, c\in [k]\rangle _{2r}^*$ be optimal for ${\xi _{r}^{\mathrm {stab}}} (K_k\star G)=k$. That is, *L* is tracial, symmetric, positive, and satisfies $L(1)~=~1$, $L(\sum _{i,c}x^i_c)=k$, and $L=0$ on $\mathscr {I}(\mathscr {H}_{K_k\star G}).$ It suffices now to construct a tracial symmetric positive linear form $\hat{L}\in \mathbb {R}\langle x_i{:}\,i\in V\rangle _{2r}^*$ such that $\hat{L}(1)=1$, $\hat{L}(\sum _{i\in V}x_i)=k$, and $\hat{L} = 0$ on $\mathscr {I}_{2r}(\mathscr {H}_G)$, since this will imply ${\xi _{r}^{\mathrm {stab}}}(G)\ge k$. For this, for any word $x_{i_1},\ldots , x_{i_t}$ with degree $1 \le t \le 2r$, we define $\hat{L}(x_{i_1},\ldots ,x_{i_t}) := \sum _{c\in [k]} L(x^{i_1}_c,\ldots , x^{i_t}_c)$, and we set $\hat{L}(1) = L(1) = 1$. Then, we have $\hat{L}(\sum _{i\in V}x_i)=k$. Moreover, one can easily check that $\hat{L}$ is indeed tracial, symmetric, positive, and vanishes on $\mathscr {I}_{2r}(\mathscr {H}_G)$. $\square$

## Footnotes

1 In fact, $C_{q}^1(\varGamma )$ consists of the correlations obtained using only local randomness.

2 To be precise, it suffices that there exists a constant $R>0$ such that $R-\sum _i x_i^2$ can be written as a sum of weighted squares $s^2 g$ with $g \in \mathscr {G} \cup \{1\}$. This is called the Archimedean condition of the quadratic module associated to $\mathscr {G}$, see Sect. 3.3.

3 The Schmidt decomposition $\psi = \sum _{i=1}^d \sqrt{\lambda _i} \ u_i \otimes v_i$ of $\psi \in \mathbb {C}^d \otimes \mathbb {C}^d$ can be viewed as the singular value decomposition $\sum _{i=1}^d \sqrt{\lambda _i} u_i v_i^*$ of the matrix $\mathrm {vec}^{-1}(\psi )$, where $\mathrm {vec}: \mathbb {C}^{d \times d} \rightarrow \mathbb {C}^d \otimes \mathbb {C}^d$ is the operation that sends $uv^*$ to $u \otimes v$.

4 We omit the explicit dependence on *i* in the integers $m_k,n_k$ to simplify the notation.
